# Matrix metalloproteinase 2‐responsive dual‐drug‐loaded self‐assembling peptides suppress tumor growth and enhance breast cancer therapy

**DOI:** 10.1002/btm2.10702

**Published:** 2024-07-17

**Authors:** Jihong Ma, Haiyan Yang, Xue Tian, Fanhu Meng, Xiaoqing Zhai, Aimei Li, Chuntao Li, Min Wang, Guohui Wang, Chunbo Lu, Jingkun Bai

**Affiliations:** ^1^ School of Clinical Medicine Shandong Second Medical University Weifang China; ^2^ Emergency Department Yantaishan Hospital Yantai China; ^3^ School of Basic Medical Sciences Shandong Second Medical University Weifang China; ^4^ School of Bioscience and Technology Shandong Second Medical University Weifang China

**Keywords:** drug delivery, enzyme, peptides, self‐assembly, tumor microenvironment

## Abstract

Conventional chemotherapeutic agents are limited by their lack of targeting and penetration and their short retention time, and chemotherapy might induce an immune suppressive environment. Peptide self‐assembly can result in a specific morphology, and the resulting morphological changes are stimuli responsive to the external environment, which is important for drug permeation and retention of encapsulated chemotherapeutic agents. In this study, a polypeptide (Pep1) containing the peptide sequences PLGLAG and RGD that is responsive to matrix metalloproteinase 2 (MMP‐2) was successfully developed. Pep1 underwent a morphological transformation from a spherical structure to aggregates with a high aspect ratio in response to MMP‐2 induction. This drug delivery system (DI/Pep1) can transport doxorubicin (DOX) and indomethacin (IND) simultaneously to target tumor cells for subsequent drug release while extending drug retention within tumor cells, which increases immunogenic cell death and facilitates the immunotherapeutic effect of CD4^+^ T cells. Ultimately, DI/Pep1 attenuated tumor‐associated inflammation, enhanced the body's immune response, and inhibited breast cancer growth by combining the actions of DOX and IND. Our research offers an approach to hopefully enhance the effectiveness of cancer treatment.

**Translational Impact Statement**

Here, we developed a drug‐loaded nanoparticle (DI/Pep1) that targets tumors while the nanoparticle transforms into aggregates with a high aspect ratio under the action of matrix metalloproteinase 2, releasing doxorubicin and prolonging the drug retention time. Importantly, DI/Pep1 increased the susceptibility to immunogenic cell death and enhanced the antitumor immune response. This development of morphologically transformable drug‐loaded nanocarriers could improve immunomodulation by prolonging the retention of chemotherapeutic agents, thereby providing pathways for the development of more effective therapeutic strategies.

## 1 INTRODUCTION

Breast cancer has overtaken lung cancer as the most frequently diagnosed cancer among women worldwide.^1^ Chemotherapy is a primary approach for treating cancer,^2^ but the majority of chemotherapeutic drugs suffer from drawbacks such as inadequate tumor cell targeting and insufficient intratumoral accumulation, which results in a range of adverse reactions and the inability to achieve the intended anticancer outcome. The dose of a drug can be increased to increase its concentration, but this also increases its toxicity to normal organs. The accurate spatial targeting of chemotherapeutics is critical. The RGD peptide targets integrin αvβ3, which is highly expressed on cancer cells, and RGD‐modified nanosystems have been demonstrated to enable active targeting of breast cancer cells.^3^

In addition to improving chemotherapy drug targeting, prolonging the retention of chemotherapy drugs in tumors is equally important. The morphology of nanocarriers plays an important role in drug encapsulation, delivery, and retention. Transformation of the morphology of the drug‐loaded system can enhance drug delivery and prolong drug retention in the tumor site.^4^, ^5^, ^6^ In particular, self‐assembled peptides can undergo morphological transformation in response to specific stimuli.^4^, ^7^, ^8^ Liu et al. designed an L‐phosphopentapeptide consisting of four L‐leucine residues and a C‐terminal L‐phosphotyrosine to self‐assemble to form micelles/nanoparticles, which were transformed into peptide nanofibers/nanoribbons induced by alkaline phosphatase. The phosphopentapeptide can selectively target the nucleus to achieve intranuclear self‐assembly and rapidly kill undifferentiated human induced pluripotent stem cells (iPSCs) while being nontoxic to normal cells.^4^ Targeting of tumor cell organelles and selective killing of tumor cells can be achieved via in situ self‐assembly or morphological transformation.^9^, ^10^, ^11^, ^12^, ^13^, ^14^ In situ enzyme‐triggered peptide self‐assembly strategies have been applied to selectively modify cell membranes or selectively degrade programmed cell death ligand 1 (PD‐L1) in tumor cells.^5^, ^15^

Self‐assembled drug‐loaded peptides can respond to microacidic pH or overexpressed enzymes in the tumor microenvironment, and the morphology of the drug vectors is altered to release chemotherapy drugs while increasing the accumulation of the drug at the tumor site.^16^, ^17^, ^18^ The concentration of matrix metalloproteinase 2 (MMP‐2) in tumor tissues exceeds that in normal tissues, which facilitates the growth, angiogenesis, invasion, and metastasis of breast cancer.^19^ A drug delivery system containing a sequence of the MMP‐2 cleavage site (PLGLAG) can eliminate the polyanionic sequence when triggered by MMP‐2 and then expose cell‐penetrating peptides for effective cellular uptake.^20^ The design of MMP‐2‐responsive drug‐loaded peptides is expected to undergo a shape shift for potential use in drug delivery.

The immune system plays a crucial role in the treatment of cancer, and cancer immunotherapy is receiving increasing attention and has promising potential for the future compared to traditional treatments.^21^, ^22^ Certain chemotherapeutic agents can lead to immune cell death (ICD) in tumor cells by inducing calreticulin (CRT) exposure.^23^, ^24^ Doxorubicin (DOX) is reportedly an ICD inducer that stimulates immune responses to necrotic cell antigens.^25^, ^26^ However, the poor accumulation of DOX in the tumor microenvironment (TME) necessitates the administration of higher doses of DOX for treatment, which can pose a significant challenge due to potential systemic toxicity.^27^ Combination therapy offers a solution by enhancing the effectiveness of a drug without the need for dose escalation.^28^ Combined therapies are becoming increasingly popular for treating cancer.^29^, ^30^, ^31^ Moreover, inflammation frequently occurs during cancer progression,^32^, ^33^ and indomethacin (IND) can alleviate inflammation by reducing factors such as interleukin 6 (IL‐6) and can work with DOX to enhance its antitumor properties, thereby increasing the overall effectiveness of treatment.^34^, ^35^, ^36^, ^37^

**SCHEME 1:**
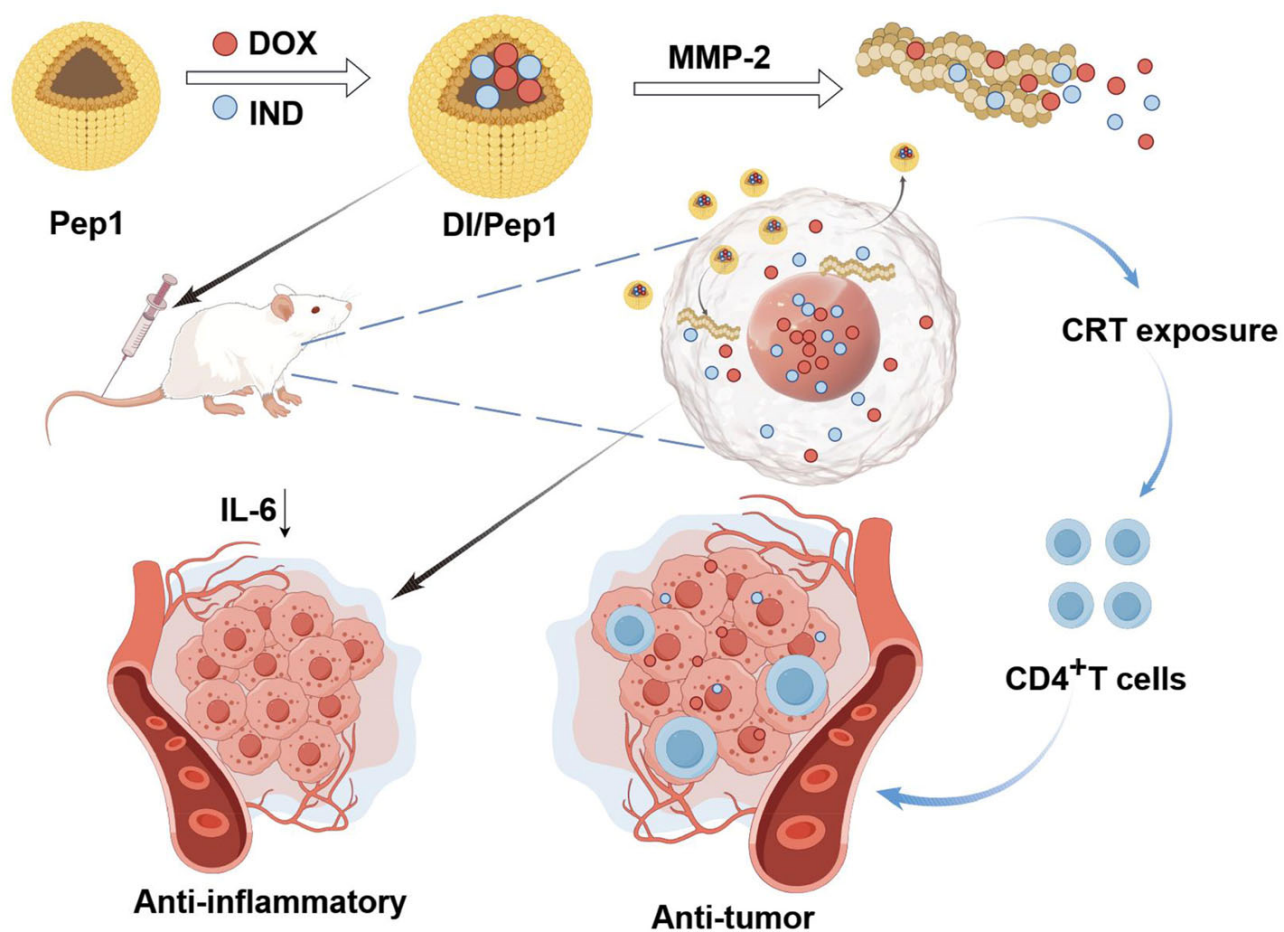
Pep1 can self‐assemble into spherical nanoparticles after the encapsulation of DOX and IND. DI/Pep1 was transformed into aggregates with a high aspect ratio under the action of MMP‐2, which prolonged the drug retention time. DI/Pep1 effectively inhibited tumor growth by promoting ICD effects and attenuating tumor inflammation (By Figdraw). DOX, doxorubicin; ICD, immune cell death; IND, indomethacin.

Hence, we designed a polypeptide with prolonged drug retention that is responsive to MMP‐2. Nanocarriers composed of self‐assembling peptides have internally hydrophobic and externally hydrophilic structures, so encapsulating the poorly soluble agents DOX and IND inside such nanoparticles is possible. The use of such an MMP‐2‐sensitive nanomedicine containing DOX and IND increases the concentration of chemotherapy drugs in tumor tissues and extends the duration of drug presence in tumor tissues. Thus, the encapsulated drugs continuously act on tumor cells, which increases the sustained ICD effect of DOX, reduces tumor‐associated inflammation, and ultimately effectively inhibits tumor growth (Scheme 1). Once applied, these enzyme‐responsive peptides encapsulating DOX and IND can greatly augment the synergistic therapeutic effects of the drugs. Moreover, this combined drug delivery approach can be expanded to reach deep‐seated tumor tissues, thereby enabling the development of a smart anticancer drug delivery system.

## 2 MATERIALS AND METHODS

### 2.1 Experimental materials

DOX was purchased from APExBIO Technology LLC (Houston, USA). IND was purchased from Aladdin. A Cell Counting Kit‐8 (CCK8) was acquired from Dojindo Laboratories (Kumamoto, Japan). MMP‐2 was obtained from Takara Bio, Inc. (Shiga, Japan). Sigma provided dimethyl sulfoxide (DMSO). Fetal bovine serum (FBS) was acquired from Gibco (Life Technologies, USA). DAPI and calcein‐AM were obtained from Beyotime Biotechnology Co. (Shanghai, China).

### 2.2 Cells and animals

MCF‐7 human breast cancer cells and human umbilical vein endothelial cells (HUVECs) were purchased from BeNa Culture Collection (BNCC). Jinan Pengyue Laboratory Animal Breeding Co. provided the BALB/c mice.

### 2.3 Peptide synthesis

QYAOBIO (ChinaPeptides Co., Ltd., Shanghai, China) synthesized the experimental peptide (Pep1) and control peptide (Pep2).

### 2.4 Drug‐loaded peptide preparation

The desired peptide (2 mg) was solubilized in 1 mL of HEPES buffer (pH 7.4) and incubated at ambient temperature. Additionally, the desired peptide (2 mg) was mixed with DOX (0.4 mg) in DMSO (10 μL) before the introduction of 990 μL of HEPES buffer. Sonication for 5 min yielded the DOX/Pep1 peptide loaded with drugs. Moreover, the desired peptide (2 mg) was dissolved in DMSO along with 0.4 mg of DOX and 0.4 mg of IND. Subsequently, 990 μL of HEPES buffer was added. The drug‐containing peptide DI/Pep1 underwent sonication for 5 min. Furthermore, 1 mL of the peptide solution was incubated at 37°C after the addition of 10 μL of 0.1 U/μL MMP‐2, 10 μL of a 1 M CaCl_2_ solution, and 30 μL of a 5 M NaCl solution to generate an enzyme‐responsive peptide solution.

### 2.5 High‐performance liquid chromatography (HPLC)

HPLC was performed according to previous experimental methods.^38^

### 2.6 Zeta potential measurements and transmission electron microscopy (TEM)

The zeta potentials of the peptide solutions and both agents loaded into the peptides were measured using a Zetasizer Nano ZS90 device manufactured by Malvern Instruments (United Kingdom). The peptide solution (30 μL) was adsorbed on a copper mesh and stained with 2.0% uranyl acetate (30 μL) for 6 min. The specimens were dried naturally in air and examined by TEM with a Hitachi HT7700 device (Tokyo, Japan).

### 2.7 In vitro drug release

DOX and IND were loaded into peptides, and their in vitro drug release characteristics were examined. To prepare peptides loaded with DI, the peptides and DI were combined at a weight ratio of 5:1:1. The reconstituted solution was added to a dialysis bag and dialyzed against 2 L of ultrapure water for 1 h. Next, 10 μL of a 10 μg/mL solution of MMP‐2 was added to the peptide solution. The dialysis bag was immersed in 25 mL of PBS and placed in a shaker at 37°C. At certain points in time, 2 mL of the liquid was removed, and 2 mL of fresh medium was added. The absorbance of the resulting solution was measured at 488 nm (DOX) and 320 nm (IND) using a UV–visible spectrophotometer (Thermo, Evolution 300, USA). A control (a peptide solution lacking MMP‐2) was also used.

### 2.8 CCK8 assay

The CCK8 assay was utilized to determine the toxicity of the peptide to HUVECs and MCF‐7 cells. After digestion and centrifugation, the cells were counted, and a 5 × 10^3^ cells/mL cell suspension was prepared. Following cell attachment, the liquid in the wells was removed, and the appropriate peptide solution (with a volume ratio of 1:1 with serum‐free medium) was added based on the experimental categorization. Following a designated period of reaction, 10 μL/100 μL of liquid CCK8 reagent was added to the wells, and the plate was placed in a 37°C oven. Subsequently, an enzyme marker (Thermo Science, USA) was used to measure the optical density of the samples at 450 nm.

### 2.9 Wound healing experiment

Cells on 6‐well plates were scratched and treated with various pharmaceutical formulations, including free DOX and IND (DI), DOX‐loaded Pep2 (DOX/Pep2), DOX‐loaded Pep1 (DOX/Pep1), DOX‐ and IND‐coloaded Pep2 (DI/Pep2), and DOX‐ and IND‐coloaded Pep1 (DI/Pep1). Cells were observed using an inverted fluorescence microscope (BX53F OLYMPUS, Japan) with an image acquisition system at 0 h and 48 h, and cell migration was documented with photographs. The migration rates of the cells were determined by calculating the ratio between the difference in the final width and the initial scratch width.

### 2.10 Transwell migration assay

After 48 h of treatment, 200 μL of a suspension of MCF‐7 cells in blank medium was added to the upper transwell chambers (8 mm, 24 wells), while 500 μL of complete medium containing 10% FBS was added to the 24‐well plates. The medium was discarded, and the cells that had not entered the edges of the transwell chambers were removed after 48 h. Then, the chambers were immersed in 4% paraformaldehyde to fix the cells. After fixation, the cells were treated with 0.1% crystal violet in the medium for 10 min and rinsed with PBS.

### 2.11 Cellular uptake

Confocal laser scanning microscopy (CLSM) and flow cytometry (FCM) were used to observe the uptake of various drug formulations by MCF‐7 cells. MCF‐7 cells were incubated with staining solution (0.1 μg/mL calcein‐AM) for 30 min, followed by 24 h of incubation at 37°C. Subsequently, the MCF‐7 cells were cocultured with the various agents for 0.25 h and 0.5 h. The cells were sequentially treated with 4% paraformaldehyde and DAPI for 15 min before being rinsed three times with PBS. CLSM was used to observe the cells. Additionally, after the same treatments, the cells were trypsinized using trypsin without EDTA. After digestion, the resulting cell suspension was centrifuged and then resuspended in PBS for quantitative analysis via FCM.

### 2.12 Cellular retention

CLSM was utilized to observe the retention of various agents by MCF‐7 cells. The cells were treated with a staining solution (0.1 μg/mL calcein‐AM) for 30 min. Ten microliters of each of the various drug formulations were added to the cells for 30 min of incubation. At 60 h and 72 h, the cells were sequentially treated with 4% paraformaldehyde and DAPI for 15 min. Finally, the cellular distributions in the different drug groups were observed and compared using CLSM.

### 2.13 Establishment of the tumor model

The Ethics Committee of Shandong Second Medical College approved all the animal experiments and methods. Female BALB/c mice, aged 6 weeks, were obtained from Peng Yue Experimental Animal Breeding Co., Ltd. 4T1 cells (1 × 10^6^ in 100 μL) were injected subcutaneously into the right axilla of the mice. The tumor volume (Tv) was calculated using the formula.

$$\mathrm{Tv}=\frac{\text{length}\times {\text{width}}^{2}}{2}.$$

### 2.14 In vivo animal imaging

When the tumor volume reached 100 mm^3^, the mice were injected with DiR, DiR/Pep2, or DiR/Pep1 via the tail vein. At various time points after injection, a small animal imaging system was used to measure the fluorescence intensities of the mice.

### 2.15 Antitumor activity in mice in vivo

When the tumor volume reached 100 mm^3^, the tumor‐bearing mice were randomized into six groups of five mice each. Tail vein injections of saline, free DI, DOX/Pep2, DOX/Pep1, DI/Pep2, or DI/Pep1 (3 mg/kg) were started on day 1, and the treatment was administered every other day thereafter. The tumor volume growth trends were plotted over 2 weeks after measuring the short and long diameters of the tumors every other day starting from day 0 to calculate the tumor volume. On day 14, the tumor‐bearing mice were euthanized to obtain the tumors and organs, including cardiac muscle, hepatic tissue, splenic tissue, pulmonary tissue, and renal tissue, which were then immersed in a solution of 4% paraformaldehyde. The tumor growth inhibition (TGI) value was calculated using the following formula:

$$\mathrm{TGI}=\left(1-\frac{\text{tumor weight in treatment group}}{\text{tumor weight in the saline group}}\right)\times 100\%.$$

### 2.16 Hematoxylin and eosin (HE) staining, immunohistochemical techniques, and immunofluorescence staining

To examine the structures of the tumor tissues and major organs of the mice at the cellular level, the mice were euthanized 24 h after the final injection. Subsequently, the tumor tissues and major organs, such as the kidneys, heart, lungs, liver, and spleen, were collected for analysis. Following fixation with 4% paraformaldehyde, the sections were stained with HE and examined, and images were acquired using a light microscope (BX53F, Olympus).

Following a series of dewaxing and hydration processes, antigen restoration, antibody incubation, color enhancement, and staining procedures, the sections were sealed using a neutral resin. Moreover, tumor tissue sections were subjected to immunofluorescence staining to detect CRT exposure and assess the extent of CD4 infiltration within the tumor. Immunohistochemical methods were used to detect the content of IL‐6.

### 2.17 Data analysis

GraphPad Prism V 8.0 software was used to process the data. Statistical comparisons were conducted using either an unmatched *t*‐test. Significant differences were determined using *t* tests.

## 3 RESULTS AND DISCUSSION

### 3.1 Preparation and characterization of the peptides

Pep1 containing the MMP‐2 substrate peptide PLGLAG and RGD targeting peptide^39^ and Pep2 without the PLGLAG peptide and RGD peptide were designed (Figures S1 and S2 in Data S1). HPLC and MS confirmed the successful synthesis of Pep1 and Pep2 with purities greater than 95% (Figures S3–S6 in Data S1).

**FIGURE 1:**
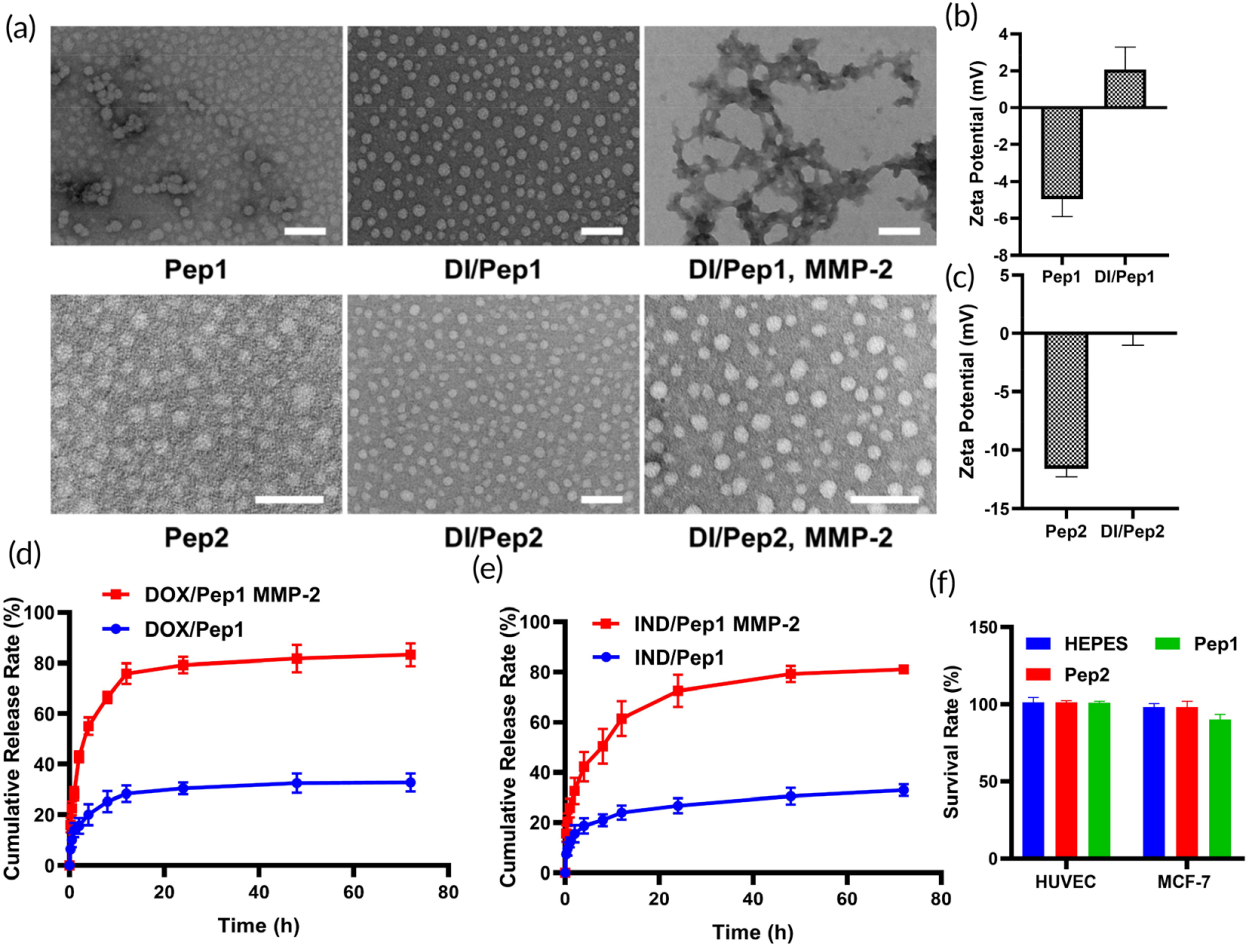
Characterization of Pep1, Pep2, and the drug‐loaded nanoparticles. (a) TEM images of the peptides, DI‐loaded peptides, and DI‐loaded peptides plus MMP‐2. (b) Zeta potentials of Pep1 and DI/Pep1. (c) Zeta potentials of Pep2 and DI/Pep2. (d) Diagram of DOX release from DOX/Pep1. (e) Diagram of IND release from IND/Pep1. (f) Toxicity of Pep1 and Pep2 to HUVECs and MCF‐7 cells in vitro. Scale bars: 100 nm. DOX, doxorubicin; HUVECs, human umbilical vein endothelial cells; IND, indomethacin; MMP‐2, matrix metalloproteinase 2; TEM, transmission electron microscopy.

Pep1 formed spherical nanoparticles measuring less than 30 nm in length in 25 mM HEPES buffer. These nanoparticles maintained their spherical shape and became slightly larger after the introduction of DOX and IND. Upon the introduction of MMP‐2 to DI/Pep1, the spherical nanoparticles aggregated, resulting in the formation of aggregates with a high aspect ratio. Pep2 and DI/Pep2 formed nanoparticles by themselves, and DI/Pep2 maintained its spherical shape even after MMP‐2 was added (Figure 1a and Figures S7–S11 in Data S1).

HPLC was used to analyze the products following the addition of MMP‐2. The intensity of the substrate peak at 14.8 min in the Pep1 sample decreased following the introduction of MMP‐2. Moreover, new product peaks emerged at 7.1 min and 17.7 min (Figure S12 in Data S1). This indicates that Pep1 is responsive to MMP‐2 and can be cleaved by MMP‐2 to generate new peptide fragments. The alteration of the peptide molecule's primary structure by the enzyme prompts a rearrangement, a transformation of the self‐assembling peptide's morphology.^40^

The surface charges of the nanoparticles were determined by measuring the zeta potentials. Both Pep1 and Pep2 were negatively charged at pH 7.4. After both drugs were loaded, the potentials of the peptides increased, resulting in DI/Pep1 having a positive charge (Figure 1b,c). A positive charge is beneficial for a drug‐loaded peptide, as it allows penetration of the TME and interaction with the tumor cell membrane.^41^

To evaluate drug release from Pep1 in the presence of MMP‐2, DI/Pep1 was prepared, and the release of DOX and IND from DI/Pep1 was evaluated separately. Over time, the drug‐loaded peptide gradually released an increasing quantity of DOX, with a relatively rapid increase of approximately 75.8% within the initial 12 h. Subsequently, the cumulative release rate stabilized and ultimately reached approximately 83.3% (Figure 1d). Moreover, the cumulative release of DOX was approximately 32.8% in the absence of MMP‐2. In the presence of MMP‐2, IND/Pep1 exhibited a cumulative release of approximately 81.1% over 72 h, whereas without MMP‐2, the cumulative release was 33.0% (Figure 1e).

Tumor cells constantly interact with the surrounding microenvironment during their physiological activities, which results in tumor tissues and normal tissues having different physiological states, and these differences, such as aberrant protein expression, provide multiple targets for tumor diagnosis and therapeutic drugs.^42^ Since the TME is rich in MMP‐2, this feature can be used to control drug release from nanoparticles mediated by MMP‐2 induction. When DI/Pep1 enters the TME, the encapsulated chemotherapeutic drugs are released after induction by MMP‐2 in the TME to exert antitumor efficacy.^43^

The in vitro cytotoxicity of the peptides was determined using CCK8 assays. After incubating the peptides with cells for 48 h, Pep1 had no apparent toxicity to HUVECs and had weak toxicity to MCF‐7 cells. Pep2 showed no significant toxicity to MCF‐7 cells or HUVECs (Figure 1f). It was thus demonstrated that both Pep1 and Pep2 cause little damage to normal cells in vitro. Under the induction of MMP‐2, Pep1 undergoes self‐assembly deformation from spherical nanoparticles to aggregates with a high aspect ratio, and the toxicity to tumor cells is enhanced.^44^

### 3.2 Drug uptake and retention in vitro

**FIGURE 2:**
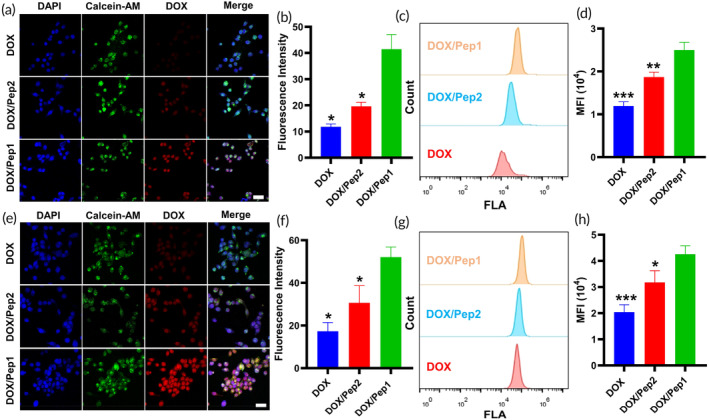
DOX uptake by MCF‐7 cells. (a) CLSM images of MCF‐7 cells treated with various formulations for 0.25 h. (b) Diagram of the quantitative fluorescence showing the effects of DOX on MCF‐7 cells at 0.25 h. (c) FCM image illustrating the impact of DOX on MCF‐7 cells at 0.25 h. (d) Quantitative FCM diagram displaying the response of MCF‐7 cells to DOX at 0.25 h. (e) CLSM images depicting the effects of DOX on MCF‐7 cells at 0.5 h. (f) Diagrams showing the measured fluorescence intensities in MCF‐7 cells cocultured with DOX for 0.5 h. (g) Diagrams of the FCM analysis of MCF‐7 cells incubated with DOX for 0.5 h. (h) Quantification of the FCM data of MCF‐7 cells cocultured with DOX for 0.5 h. Scale bars: 50 μm. *p < 0.05, **p < 0.01 and ***p < 0.001, compared with the DOX/Pep1 group. CLSM, confocal laser scanning microscopy; DOX, doxorubicin; FCM, flow cytometry.

Next, we qualitatively assessed the uptake of DOX, DOX/Pep2, and DOX/Pep1 by MCF‐7 cells using CLSM. The fluorescence of DOX/Pep1 was detected inside the cells after incubation for 0.25 h, and the fluorescence intensity in this group was significantly greater than that in both the DOX and DOX/Pep2 groups, suggesting that the MCF‐7 cells took up more DOX/Pep1 during the same incubation period (Figure 2a). The semiquantitative results of red fluorescence intensity from the CLSM observations were in agreement with the results (Figure 2b). FCM was used to further analyze the fluorescence intensities in cells coincubated with different preparations, and the fluorescence intensity in the DOX/Pep1 group was greater than that in the DOX/Pep2 and free DOX groups (Figure 2c,d). As seen from the CLSM images (Figure 2e) and the semiquantitative fluorescence analysis (Figure 2f), as well as the FCM results (Figure 2g,h), after 0.5 h, the DOX/Pep1 group was still the strongest. The RGD‐modified peptide nanoparticles easily targeted the vicinity of the tumor cells.^45^ As the number of drug‐loaded peptide nanoparticles near the tumor cells increased, the number of DOX‐encapsulating nanoparticles absorbed by the MCF‐7 cells eventually increased, and the RGD motif enhanced tumor penetration.^46^

**FIGURE 3:**
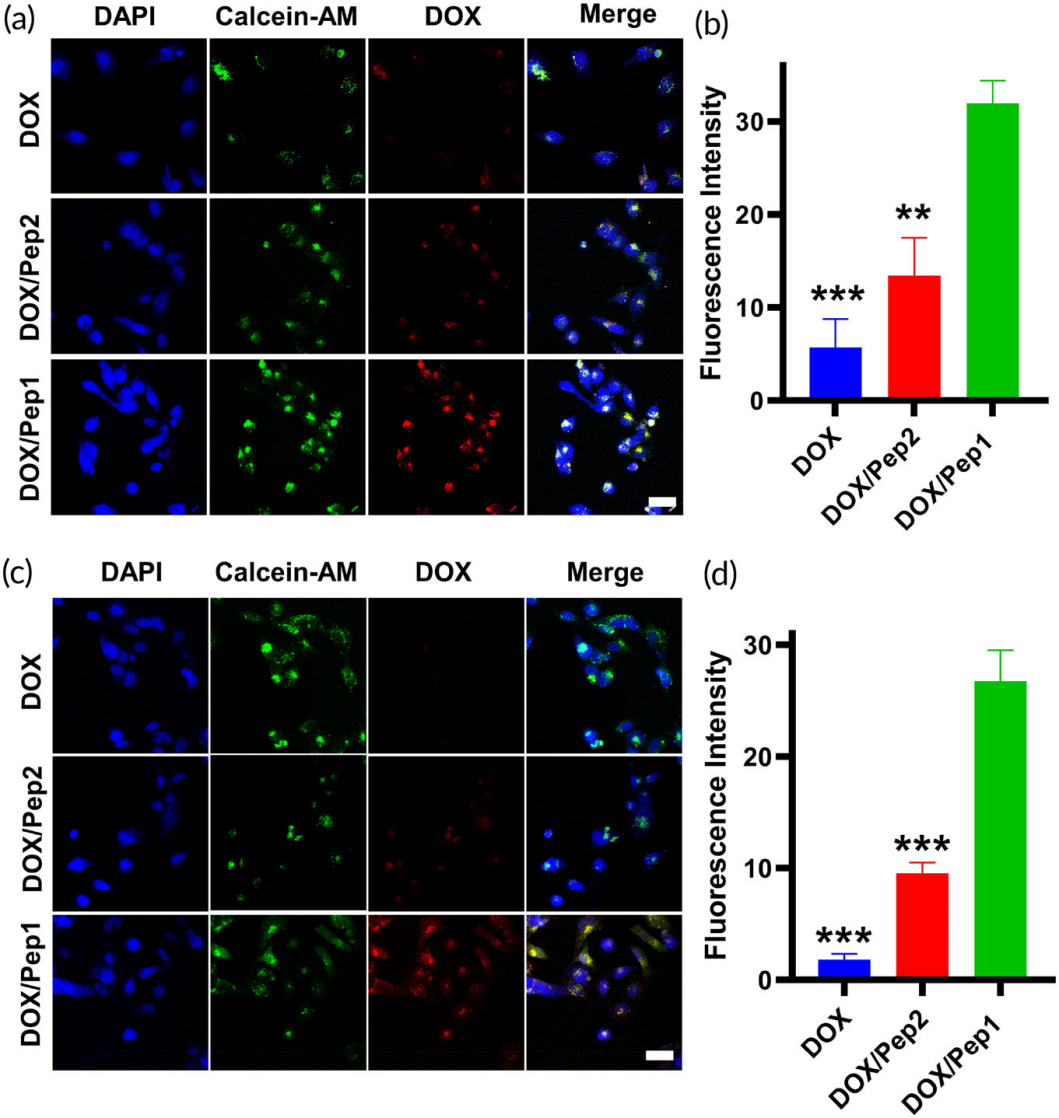
Retention of DOX by MCF‐7 cells. (a) CLSM images of MCF‐7 cells treated with various formulations for 60 h. (b) Quantitative fluorescence images of MCF‐7 cells cocultured with DOX for 60 h. (c) CLSM images of MCF‐7 cells incubated with DOX for 72 h. (d) Quantitative fluorescence images of MCF‐7 cells treated with DOX for 72 h. **p < 0.01 and ***p < 0.001, compared with the DOX/Pep1 group. Scale bars: 50 μm. CLSM, confocal laser scanning microscopy; DOX, doxorubicin.

To further examine the retention of various drug preparations in cancer cells, the retention of DOX in cells cocultivated with DOX, DOX/Pep2, and DOX/Pep1 was observed for 60 h and 72 h using CLSM. A comparison of the CLSM images after different incubation periods revealed that the red fluorescence of free DOX was almost undetectable after 60 h, and little free DOX was retained in the cells. Both the DOX/Pep2 and DOX/Pep1 groups exhibited significant red fluorescence, with the DOX/Pep1 group displaying stronger fluorescence intensity than the DOX/Pep2 group. This indicates that DOX/Pep1 was more effectively retained within these cells (Figure 3a). Semiquantitative fluorescence analysis revealed that the fluorescence intensity in the DOX/Pep1 group was considerably greater than that in the DOX/Pep2 and DOX groups (Figure 3b). After 72 h of culture, a significant amount of red fluorescence was observed in the cells in the DOX/Pep1 group compared with the free DOX and DOX/Pep2 groups (Figure 3c,d). The CLSM images and semiquantitative fluorescence data indicated that DOX retention was greater in the DOX/Pep1 group than in the other groups. This may be because this drug‐loaded peptide can transform into aggregates with a high aspect ratio in the presence of MMP‐2,^47^, ^48^, ^49^ which facilitates the prolongation of the retention time of drug‐carrying nanoformulations in cells.

### 3.3 Wound healing and transwell assays

**FIGURE 4:**
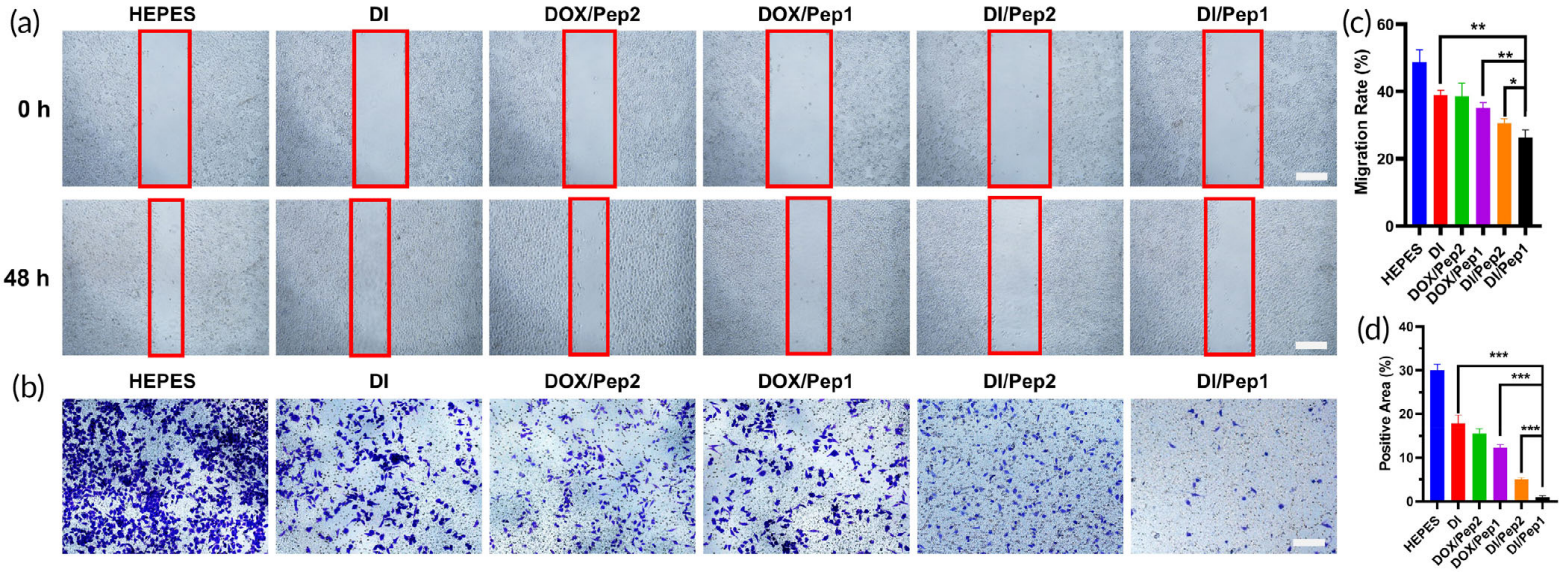
Impact of the drug‐loaded peptide on the migration of MCF‐7 cells. (a) Diagram illustrating the lateral migration (healing) of MCF‐7 cells after treatment with drug‐loaded peptides. Scale bars: 500 μm. (b) Transwell assay results with the drug‐loaded peptides. Scale bar: 200 μm. (c) Quantitative results from the scratch healing assay. (d) Quantitative image of the transwell assay data. *p < 0.05, **p < 0.01 and ***p < 0.001.

To evaluate wound healing, an experiment was conducted to determine how different drug formulations affect the lateral migration ability of MCF‐7 cells. The findings indicated that DOX/Pep1 and DOX/Pep2 hindered the movement of some MCF‐7 cells. Furthermore, the inhibition of migration was enhanced by the addition of IND, and the inhibitory effect of DI/Pep1 was the most significant. This finding suggested that IND can work in combination with DOX to further suppress the migration of MCF‐7 cells (Figure 4a).

To examine the impact of the drug‐loaded peptides on the longitudinal migration capability of MCF‐7 cells, additional transwell experiments were conducted. In the blank control group, many MCF‐7 cells exhibited robust longitudinal migration toward the lower compartment. However, after 48 h of treatment with DOX/Pep1 and DOX/Pep2, the number of MCF‐7 cells migrating to the lower compartment was notably decreased. Following 48 h of intervention with DI/Pep1 and DI/Pep2, there was a further decrease in the number of cells migrating to the lower compartment, exhibiting significant disparities compared to the blank control group. Notably, DI/Pep1 demonstrated the most pronounced inhibitory effect (Figure 4b). After encapsulating IND with DOX, the inhibition of cell migration was more obvious, as shown in the semiquantitative graph (Figure 4c,d). This finding demonstrated that IND has the potential to enhance the inhibitory effect of DOX on the migration of MCF‐7 cells when used in combination with DOX.

### 3.4 Drug uptake and retention in vivo

**FIGURE 5:**
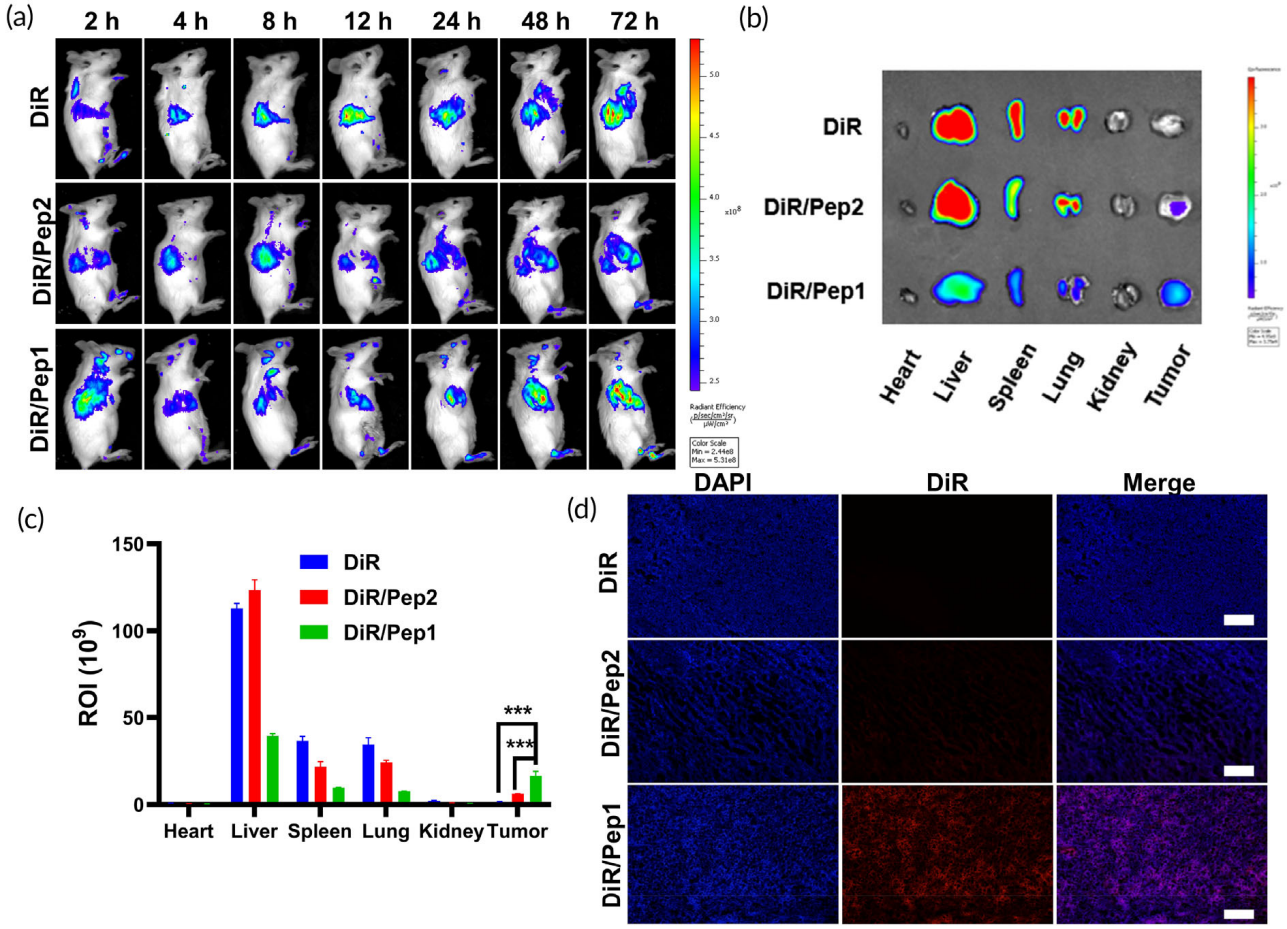
Distribution of the drug‐loaded peptides in mice. (a) Fluorograms of live mice were captured at various time intervals. (b) Fluorescence images of major organs and tumors of mice after 72 h and (c) quantitative assessment of fluorescence intensity, ***p < 0.001. (d) Microscopy images of frozen tumor sections. Scale bars: 200 μm.

Different groups of mice were randomized into different groups of breast cancer models. The fluorescence imaging of nanomaterials can be achieved by preparing nanocarriers that carry the fluorescent dye DiR. Mice were injected with free DiR, DiR/Pep2, or DiR/Pep1 through the tail vein. More than 4 h after injection, the brightness of DiR/Pep1 at the tumor location was considerably greater than that of both DiR/Pep2 and DiR. Furthermore, 24 h after injection, the DiR/Pep1 group exhibited concentrated fluorescence at the tumor site, suggesting effective tumor targeting by the nanoparticles in the treated mice (Figure 5a). Seventy‐two hours after injection, there was also a stronger DiR fluorescence signal in the DI/Pep1‐treated mice than in the DiR/Pep2‐treated mice. This suggests that DiR/Pep1 enhances drug accumulation at the tumor site, which may be related to the morphological transformation of DiR/Pep1.^43^, ^48^ To visually analyze the drug targeting and distribution in tissues, bar graphs of the intensity of fluorescence were generated (Figure 5b,c). The mice in the DiR/Pep1 group exhibited the most prominent fluorescence signals in their tumors, which were 8.8 times more intense than those in the free DiR group and 2.9 times greater than those in the DiR/Pep2 group. In the mice, DiR/Pep1 had a greater distribution in the tumors than free DiR and DiR/Pep2, suggesting that Pep1 improves drug targeting and prolongs drug retention at the tumor site (Figure 5d). In contrast to regular tissues, solid tumor tissues exhibit many blood vessels, wider interstitial spaces within the vessel wall, compromised structural integrity, and an absence of lymphatic return. These factors make the accumulation of nanoparticles in tumor tissue easier. The increase in DiR tumor accumulation suggests that after injection via the tail vein, DiR/Pep1 has a remarkable ability to target the tumor and exhibits prolonged retention and accumulation in tumor tissues, thereby effectively extending the duration of drug action.

### 3.5 Antitumor effect of DI/Pep1 in vivo

**FIGURE 6:**
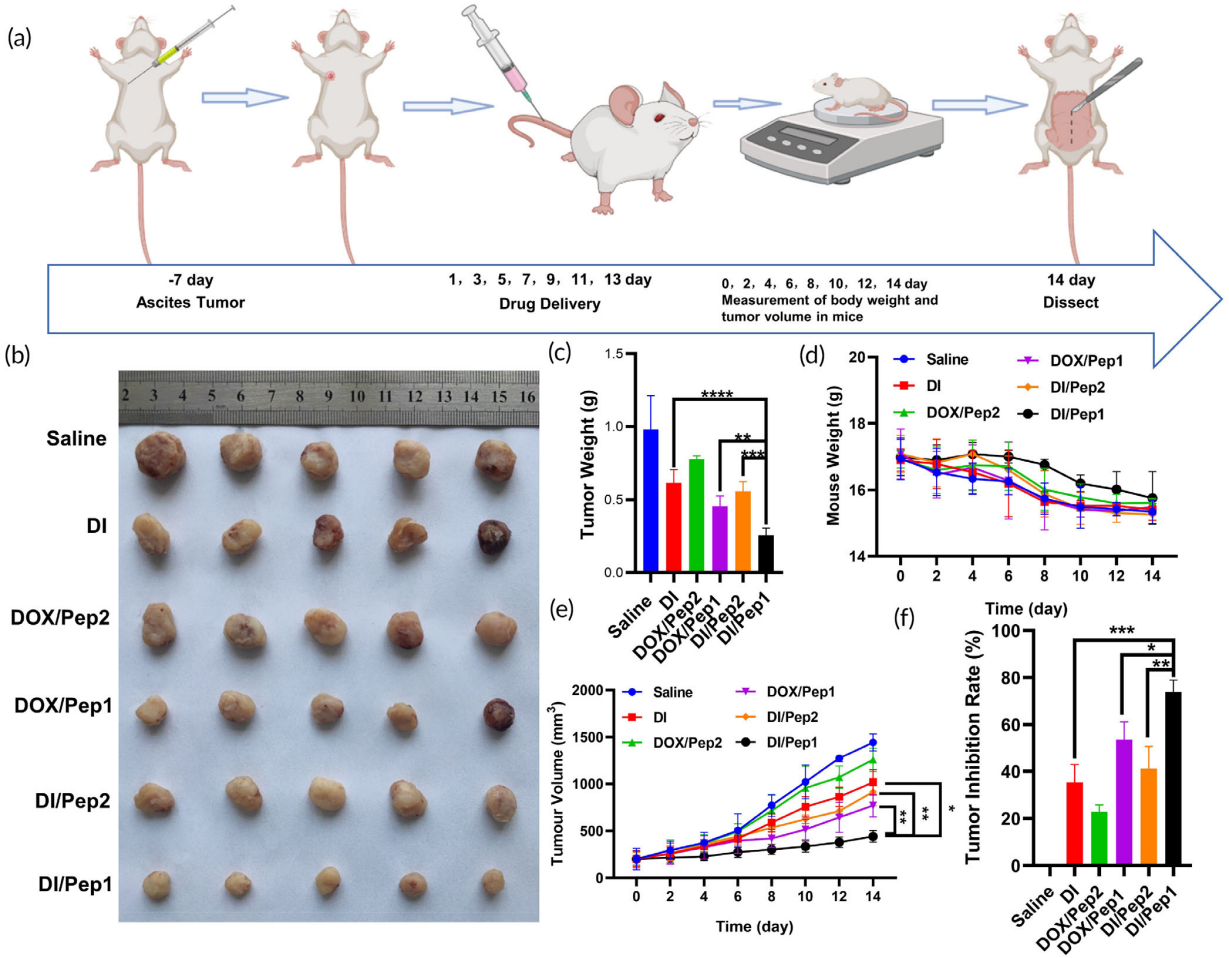
In vivo experiments were conducted with mice to test the effectiveness of the nanomaterials against tumors. (a) Flowchart depicting the in vivo anticancer process in mice (generated with BioRender). (b) Pictures of mouse tumors. (c) Statistical analysis of tumor weight. (d) Changes in mouse body weight. (e) Statistical analysis of tumor volume. (f) The histogram of TGI. *p < 0.05, **p < 0.01, ***p < 0.001 and ****p < 0.0001. TGI, tumor growth inhibition.

In vivo, 4T1 tumor‐bearing mice were used to investigate the antitumor effects of various drug formulations (Figure 6a). The therapeutic effects of various drug formulations (saline, free DI, DOX/Pep2, DOX/Pep1, DI/Pep2, and DI/Pep1) against tumors in live mice were examined following injections into the tail vein. After treatment was completed, the mice in the DI/Pep1 group had the smallest tumors and the smallest change in body weight compared to those in the other groups (Figure 6b–e). The tumor suppression rate in the DI/Pep1 group was significantly greater than that in the DI, DOX/Pep1, and DI/Pep2 groups (Figure 6f). These results indicate that DI/Pep1 inhibited the growth of tumors more effectively than the other treatments.^50^, ^51^

**FIGURE 7:**
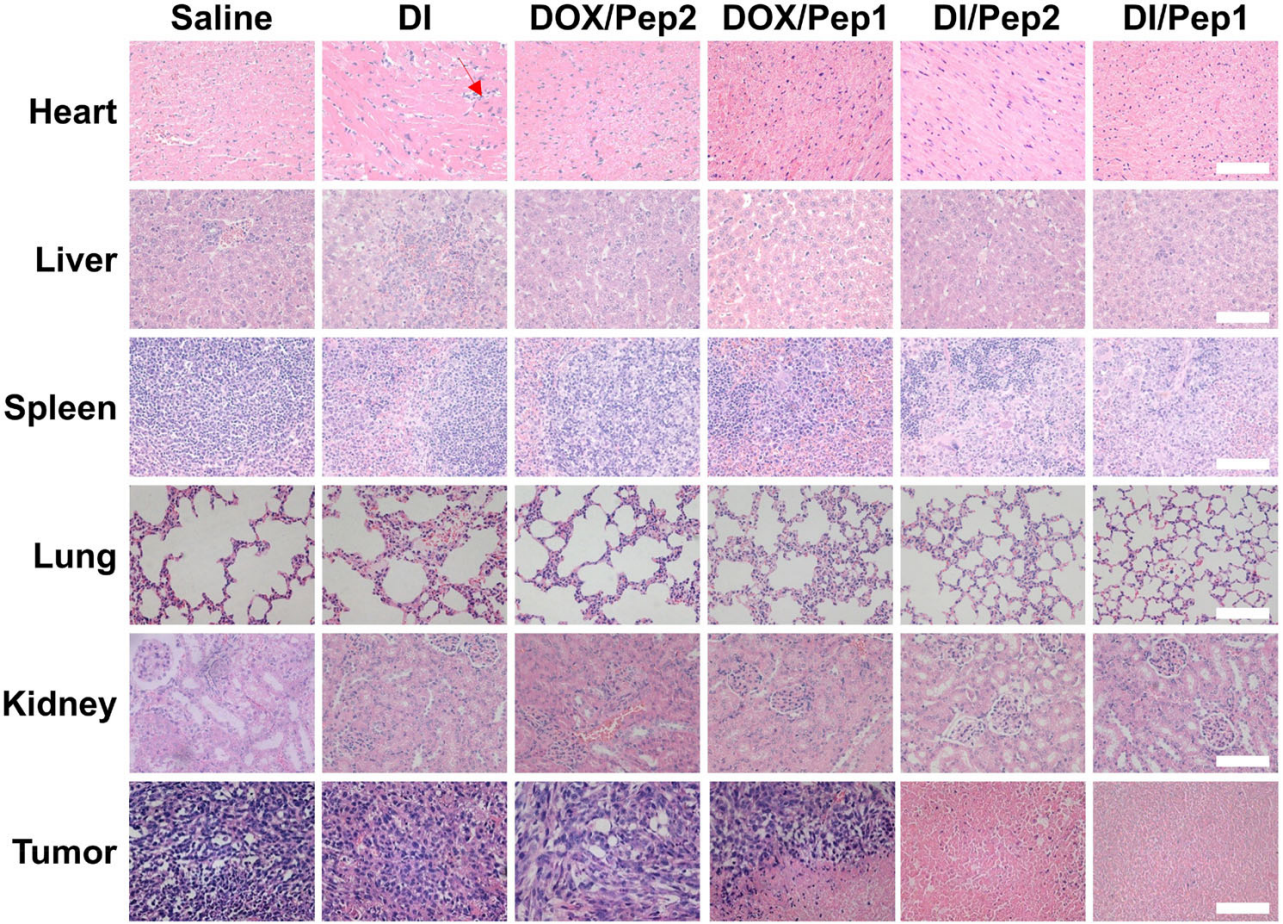
Representative images of HE‐stained sections of major organs and tumors. Scale bars: 100 μm. HE, hematoxylin and eosin.

Studies have shown that DOX has the potential to cause severe systemic toxicity, such as cardiac damage, when it is administered repeatedly at high doses.^52^, ^53^, ^54^ The results of HE staining showed that free DI caused some damage to cardiomyocytes, as some cardiomyocyte fibers were disrupted. In contrast, there was no significant structural damage to the heart tissue after the drugs were encapsulated by the nanocarriers (Figure 7).

**FIGURE 8:**
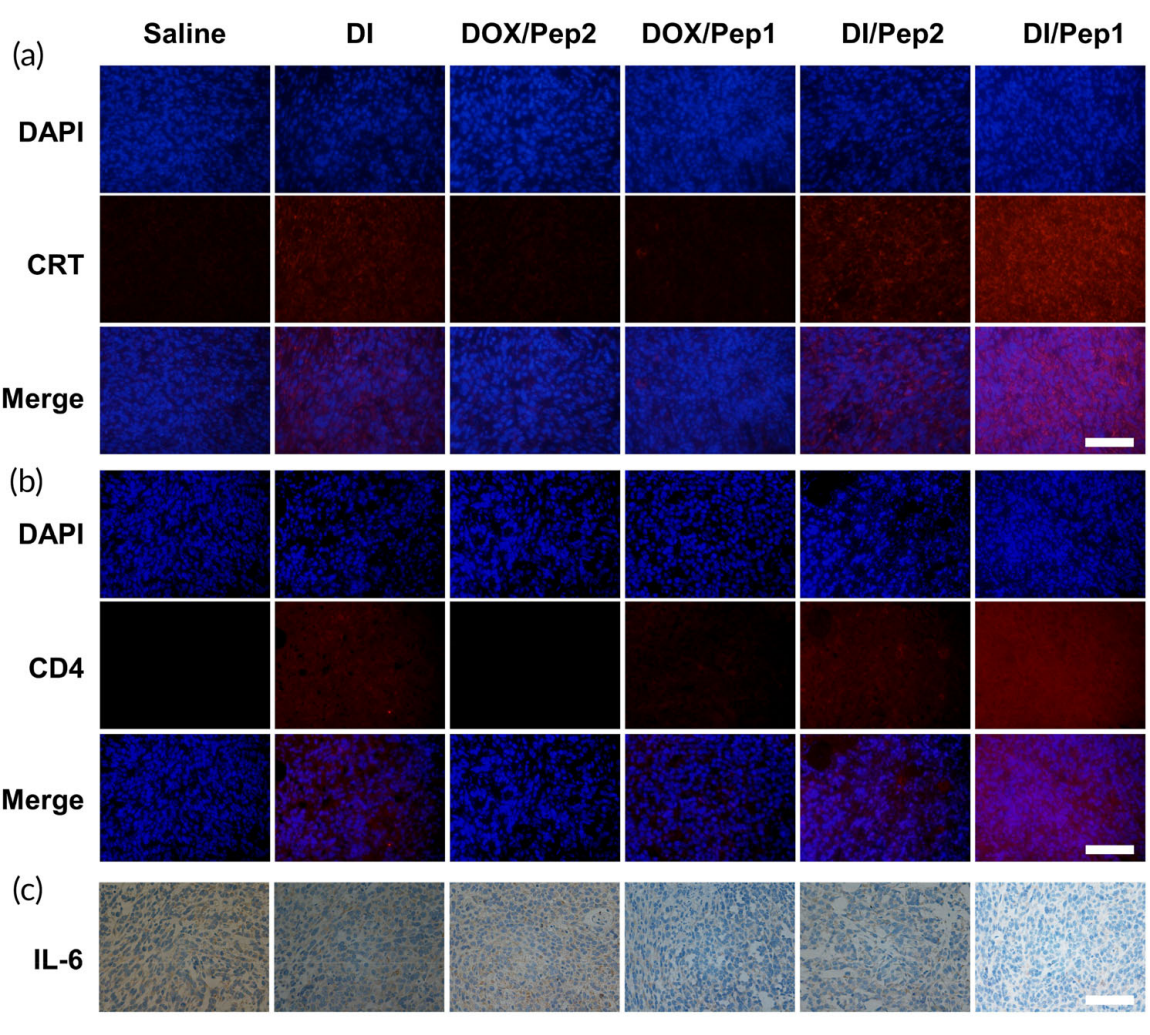
Mouse tumor section images. Tumor sections were processed for (a) CRT, (b) CD4 immunofluorescence, and (c) IL‐6 immunohistochemistry. Scale bars: 50 μm. CRT, calreticulin.

Studies have shown that ICD inducers such as DOX can effectively induce the translocation of CRT to the cell surface prior to apoptosis.^55^ Therefore, we investigated CRT exposure in tumor tissue. The results showed that the DI/Pep1 group had the highest rate of CRT exposure among the groups. Notably, the DI group had lower CRT exposure due to the poor water solubility of the drugs, and DOX/Pep2 and DI/Pep2 were not responsive to MMP‐2 and therefore could not accumulate effectively in tumor tissues (Figure 8a).

The immune status in the TME can be evaluated using CD4 expression, and there is a positive correlation between alterations in CD4 levels and CD4^+^ T‐cell infiltration within the TME.^56^ CD4^+^ T cells play a significant role in antitumor immunity.^57^ The results showed that there were the most CD4^+^ T cells and the most extensive infiltration in the tumor sections in the DI/Pep1 group, surpassing the other groups that received different treatments (Figure 8b). This indicates that this strategy of administration can improve the immunogenicity of tumor cells and facilitate immune system identification, infiltration, and eradication of tumor tissues.^58^

Overexpression of IL‐6 contributes to tumor aggressiveness.^34^ Oncogenes associated with the inflammatory microenvironment produce IL‐6, which promotes the development of breast cancer.^59^, ^60^, ^61^ The DI/Pep1 group exhibited the lowest rate of IL‐6 positivity according to IL‐6 immunohistochemical analysis, while the dual‐drug group demonstrated decreased IL‐6 positivity compared to the single drug group owing to IND's ability to mitigate inflammation and consequently diminish IL‐6 levels (Figure 8c).

Small animal images confirmed that DI/Pep1 can target tumors and allow drugs to be retained for an extended period. Studies have demonstrated that particles smaller than 50 nm exhibit enhanced tumor permeability as a result of decreased diffusion barriers.^62^, ^63^, ^64^ The conversion of this peptide nanocarrier into aggregates with a high aspect ratio very effectively increased the drug concentration.^38^ In addition to in vivo anticancer tests in mice, the peptide, which carried both DOX and IND, successfully impeded tumor growth via this drug delivery approach. This strategy can effectively suppress tumor growth, reduce tumor‐associated inflammation, and improve immune function, as demonstrated by HE staining, immunohistochemistry, and immunofluorescence.

## 4 DISCUSSION

Here, we designed Pep1 with an RGD sequence and demonstrated that it can undergo morphological transformation in the presence of MMP‐2. The drug‐loaded peptide DI/Pep1 can actively target tumor tissues and undergo a transformation from a spherical shape to an aggregate shape with a high aspect ratio induced by MMP‐2 in tumor tissues. DI/Pep1 encapsulates the traditional chemotherapeutic agent DOX, which can cause ICD in tumor cells. Immunohistochemical staining of tumor tissue demonstrated that DI/Pep1 enhanced the expression of CD4 and improved immune function. IND has the potential to reduce tumor‐associated inflammation. Immunohistochemical staining of tumor tissue indicated that DI/Pep1 reduced the expression of the inflammatory factor IL‐6. Both DOX and IND act synergistically, and DI/Pep1 exhibited a more pronounced antitumor effect than did DOX/Pep1.

In vivo and in vitro assays demonstrated that drug‐loaded Pep1 retained more drugs in the tumor than did the other groups for the same duration of time. Previous studies have demonstrated that peptides containing RGD sequences can effectively target tumor tissues.^3^ In the TME, the morphology of peptides transforms from spherical to nanofibrils or high aspect ratio aggregates when exposed to low pH or overexpressed enzymes. This prolongs the retention time of the drug in the tumor tissues, increases the aggregation of the drug in the tumor site, and improves the antitumor effect.^16^, ^17^, ^18^

A tumor‐bearing mouse model was used in this study. Mouse models are commonly used for preclinical validation of tumor therapies, but these models differ significantly from actual clinical patients, particularly because the immune system of mice differs from that of humans in many ways. In addition, although complex nanodelivery systems can better fulfill different needs, this may increase the complexity of production during the manufacturing process. Therefore, there is still a need to develop simple and effective nanodelivery systems to achieve industrialized production, which requires a significant amount of research and effort.^65^

## 5 CONCLUSION

In this work, we constructed an RGD‐modified, self‐assembling peptide system responsive to the enzyme MMP‐2 that was coloaded with DOX and IND. The tumor cell targeting properties of DI/Pep1 nanoparticles enhanced drug uptake by tumor cells. DI/Pep1 is cleaved by MMP‐2 in the TME and transforms into aggregates with a high aspect ratio, thus controlling the drug release rate and prolonging the retention time of the drug in tumor tissues and enhancing site specificity and efficacy. In particular, in in vivo experiments, the peptide enhanced the antitumor effect of DOX through the combination of IND, decreased IL‐6 expression, increased CRT and CD4 levels, attenuated tumor‐associated inflammation, and enhanced immunity, which effectively inhibited breast cancer growth.

## AUTHOR CONTRIBUTIONS

**Jihong Ma:** Data curation; investigation; methodology; writing – original draft. **Haiyan Yang:** Data curation; formal analysis. **Xue Tian:** Data curation; formal analysis. **Fanhu Meng:** Formal analysis; investigation. **Xiaoqing Zhai:** Formal analysis; investigation. **Aimei Li:** Investigation. **Chuntao Li:** Investigation. **Min Wang:** Supervision. **Guohui Wang:** Supervision. **Chunbo Lu:** Supervision. **Jingkun Bai:** Conceptualization; funding acquisition; supervision; writing – review and editing.

## CONFLICT OF INTEREST STATEMENT

The authors declare no conflicts of interest.

## Supporting information


**Data S1.** Supporting information.

## Data Availability

The data that support the findings of this study are available from the corresponding author upon reasonable request.

## References

[1] Sung H , Ferlay J , Siegel RL , et al. Global cancer statistics 2020: GLOBOCAN estimates of incidence and mortality worldwide for 36 cancers in 185 countries. CA Cancer J Clin. 2021;71(3):209‐249. 33538338 10.3322/caac.21660

[2] Liu J , Huang J , Zhang L , Lei J . Multifunctional metal‐organic framework heterostructures for enhanced cancer therapy. Chem Soc Rev. 2021;50(2):1188‐1218. 33283806 10.1039/d0cs00178c

[3] Sofias AM , Toner YC , Meerwaldt AE , et al. Tumor targeting by α_v_β_3_‐integrin‐specific lipid nanoparticles occurs via phagocyte hitchhiking. ACS Nano. 2020;14(7):7832‐7846. 32413260 10.1021/acsnano.9b08693 PMC7392528

[4] Liu S , Zhang Q , Shy AN , et al. Enzymatically forming intranuclear peptide assemblies for selectively killing human induced pluripotent stem cells. J Am Chem Soc. 2021;143(38):15852‐15862. 34528792 10.1021/jacs.1c07923 PMC8588069

[5] Ding Y , Zheng D , Xie L , et al. Enzyme‐instructed peptide assembly favored by preorganization for cancer cell membrane engineering. J Am Chem Soc. 2023;145(8):4366‐4371. 36669158 10.1021/jacs.2c11823

[6] Wang K , Zhang X , Ye H , et al. Biomimetic nanovaccine‐mediated multivalent IL‐15 self‐transpresentation (MIST) for potent and safe cancer immunotherapy. Nat Commun. 2023;14(1):6748. 37875481 10.1038/s41467-023-42155-z PMC10598200

[7] Jia HR , Zhu YX , Liu X , et al. Construction of dually responsive nanotransformers with nanosphere‐nanofiber‐nanosphere transition for overcoming the size paradox of anticancer nanodrugs. ACS Nano. 2019;13(10):11781‐11792. 31553562 10.1021/acsnano.9b05749

[8] Fan Y , Li XD , He PP , et al. A biomimetic peptide recognizes and traps bacteria in vivo as human defensin‐6. Sci Adv. 2020;6(19):eaaz4767. 32494712 10.1126/sciadv.aaz4767 PMC7209993

[9] Yi M , Wang F , Tan W , Hsieh JT , Egelman EH , Xu B . Enzyme responsive rigid‐rod aromatics target “undruggable” phosphatases to kill cancer cells in a mimetic bone microenvironment. J Am Chem Soc. 2022;144(29):13055‐13059. 35849554 10.1021/jacs.2c05491 PMC9339482

[10] Tan W , Zhang Q , Quiñones‐Frías MC , et al. Enzyme‐responsive peptide thioesters for targeting Golgi apparatus. J Am Chem Soc. 2022;144(15):6709‐6713. 35404599 10.1021/jacs.2c02238 PMC9069992

[11] Jana B , Jin S , Go EM , et al. Intra‐lysosomal peptide assembly for the high selectivity index against cancer. J Am Chem Soc. 2023;145(33):18414‐18431. 37525328 10.1021/jacs.3c04467

[12] Zhang L , Jing D , Jiang N , et al. Transformable peptide nanoparticles arrest HER2 signalling and cause cancer cell death in vivo. Nat Nanotechnol. 2020;15(2):145‐153. 31988501 10.1038/s41565-019-0626-4 PMC7147967

[13] Chen Z , Zhang K , Fan J , et al. In situ construction of ligand nano‐network to integrin α_v_β_3_ for angiogenesis inhibition. Chin Chem Lett. 2020;31(12):3107‐3112.

[14] Yang PP , Zhang K , He PP , et al. A biomimetic platelet based on assembling peptides initiates artificial coagulation. Sci Adv. 2020;6(22):eaaz4107. 32766439 10.1126/sciadv.aaz4107 PMC7385434

[15] Wang Y , Li X , Zheng D , Chen Y , Zhang Z , Yang Z . Selective degradation of PD‐L1 in cancer cells by enzyme‐instructed self‐assembly. Adv Funct Mater. 2021;31(45):2102505.

[16] Yuan X , Liu X , Li H , et al. pH‐triggered transformable peptide nanocarriers extend drug retention for breast cancer combination therapy. Adv Healthc Mater. 2024;13:2400031 Online ahead of print. 10.1002/adhm.202400031 38588449

[17] Liu Y , Liu Y , Sun X , Wang Y , du C , Bai J . Morphologically transformable peptide nanocarriers coloaded with doxorubicin and curcumin inhibit the growth and metastasis of hepatocellular carcinoma. Mater Today Bio. 2024;24:100903. 10.1016/j.mtbio.2023.100903 PMC10733681 38130427

[18] Sun X , Gao W , Liu Y , et al. pH‐responsive morphology shifting peptides coloaded with paclitaxel and sorafenib inhibit angiogenesis and tumor growth. Mater Des. 2024;238:112619.

[19] Muniz‐Bongers LR , McClain CB , Saxena M , Bongers G , Merad M , Bhardwaj N . MMP2 and TLRs modulate immune responses in the tumor microenvironment. JCI Insight. 2021;6(12):e144913. 34032639 10.1172/jci.insight.144913 PMC8262464

[20] Yin J , Liu D , Bao L , et al. Tumor targeting and microenvironment‐responsive multifunctional fusion protein for pro‐apoptotic peptide delivery. Cancer Lett. 2019;452:38‐50. 30904618 10.1016/j.canlet.2019.03.016

[21] Zhang J , Sun X , Zhao X , et al. Combining immune checkpoint blockade with ATP‐based immunogenic cell death amplifier for cancer chemo‐immunotherapy. Acta Pharm Sin B. 2022;12(9):3694‐3709. 36176905 10.1016/j.apsb.2022.05.008 PMC9513492

[22] Zhang S , Zhang Y , Feng Y , et al. Biomineralized two‐enzyme nanoparticles regulate tumor glycometabolism inducing tumor cell pyroptosis and robust antitumor immunotherapy. Adv Mater. 2022;34(50):e2203851. 10.1002/adma.202206851 36193764

[23] Kim DY , Pyo A , Yun M , et al. Imaging calreticulin for early detection of immunogenic cell death during anticancer treatment. J Nucl Med. 2021;62(7):956‐960. 33509975 10.2967/jnumed.120.245290 PMC8882882

[24] Jeon J , Yoon B , Dey A , et al. Self‐immolative polymer‐based immunogenic cell death inducer for regulation of redox homeostasis. Biomaterials. 2023;295:122064. 36827894 10.1016/j.biomaterials.2023.122064

[25] Yang S , Shim MK , Kim WJ , et al. Cancer‐activated doxorubicin prodrug nanoparticles induce preferential immune response with minimal doxorubicin‐related toxicity. Biomaterials. 2021;272:120791. 33831739 10.1016/j.biomaterials.2021.120791

[26] Li Q , Liu J , Fan H , et al. IDO‐inhibitor potentiated immunogenic chemotherapy abolishes primary tumor growth and eradicates metastatic lesions by targeting distinct compartments within tumor microenvironment. Biomaterials. 2021;269:120388. 33172606 10.1016/j.biomaterials.2020.120388

[27] Shafei A , El‐Bakly W , Sobhy A , et al. A review on the efficacy and toxicity of different doxorubicin nanoparticles for targeted therapy in metastatic breast cancer. Biomed Pharmacother. 2017;95:1209‐1218. 28931213 10.1016/j.biopha.2017.09.059

[28] Wei G , Wang Y , Yang G , Wang Y , Ju R . Recent progress in nanomedicine for enhanced cancer chemotherapy. Theranostics. 2021;11(13):6370‐6392. 33995663 10.7150/thno.57828 PMC8120226

[29] Amaldoss MJN , Yang J‐L , Koshy P , Unnikrishnan A , Sorrell CC . Inorganic nanoparticle‐based advanced cancer therapies: promising combination strategies. Drug Discov Today. 2022;27(12):103386. 36182068 10.1016/j.drudis.2022.103386

[30] Plana D , Palmer AC , Sorger PK . Independent drug action in combination therapy: implications for precision oncology. Cancer Discov. 2022;12(3):606‐624. 34983746 10.1158/2159-8290.CD-21-0212 PMC8904281

[31] Pomeroy AE , Schmidt EV , Sorger PK , Palmer AC . Drug independence and the curability of cancer by combination chemotherapy. Trends Cancer. 2022;8(11):915‐929. 35842290 10.1016/j.trecan.2022.06.009 PMC9588605

[32] Todoric J , Antonucci L , Karin M . Targeting inflammation in cancer prevention and therapy. Cancer Prev Res. 2016;9(12):895‐905. 10.1158/1940-6207.CAPR-16-0209 PMC5142754 27913448

[33] van der Willik KD , Koppelmans V , Hauptmann M , Compter A , Ikram MA , Schagen SB . Inflammation markers and cognitive performance in breast cancer survivors 20 years after completion of chemotherapy: a cohort study. Breast Cancer Res. 2018;20(1):135. 30442190 10.1186/s13058-018-1062-3 PMC6238315

[34] Rašková M , Lacina L , Kejík Z , et al. The role of IL‐6 in cancer cell invasiveness and metastasis—overview and therapeutic opportunities. Cells. 2022;11(22):3698. 36429126 10.3390/cells11223698 PMC9688109

[35] Rincon M . Interleukin‐6: from an inflammatory marker to a target for inflammatory diseases. Trends Immunol. 2012;33(11):571‐577. 22883707 10.1016/j.it.2012.07.003

[36] Mei L , He S , Liu Z , Xu K , Zhong W . Co‐assembled supramolecular hydrogels of doxorubicin and indomethacin‐derived peptide conjugates for synergistic inhibition of cancer cell growth. Chem Commun. 2019;55(30):4411‐4414. 10.1039/c9cc00590k 30916078

[37] Katz IM . Indomethacin. Ophthalmology. 1981;88(5):455‐458. 7267020 10.1016/s0161-6420(81)35004-0

[38] Hong Z , Sun X , Sun X , et al. Enzyme‐induced morphological transformation of drug carriers: implications for cytotoxicity and the retention time of antitumor agents. Mater Sci Eng C Mater Biol Appl. 2021;129:112389. 34579908 10.1016/j.msec.2021.112389

[39] Jin Q , Deng Y , Chen X , Ji J . Rational design of cancer nanomedicine for simultaneous stealth surface and enhanced cellular uptake. ACS Nano. 2019;13(2):954‐977. 30681834 10.1021/acsnano.8b07746

[40] Cao J , Yuan X , Sun X , et al. Matrix metalloproteinase‐2‐induced morphologic transformation of self‐assembled peptide nanocarriers inhibits tumor growth and metastasis. ACS Materials Lett. 2023;5(3):900‐908.

[41] Fan Z , Ji Z , Zhang F , et al. Charge reversal hairpin peptide modified synergy therapeutic nanoplatforms for tumor specific drug shuttling. Biomater Sci. 2022;10(17):4889‐4901. 35861355 10.1039/d2bm00817c

[42] Jin MZ , Jin WL . The updated landscape of tumor microenvironment and drug repurposing. Signal Transduct Target Ther. 2020;5(1):166. 32843638 10.1038/s41392-020-00280-x PMC7447642

[43] Gong Z , Zhou B , Liu X , et al. Enzyme‐induced transformable peptide nanocarriers with enhanced drug permeability and retention to improve tumor nanotherapy efficacy. ACS Appl Mater Interfaces. 2021;13(47):55913‐55927. 34784165 10.1021/acsami.1c17917

[44] Gong Z , Shi Y , Tan H , et al. Plasma amine oxidase‐induced nanoparticle‐to‐nanofiber geometric transformation of an amphiphilic peptide for drug encapsulation and enhanced bactericidal activity. ACS Appl Mater Interfaces. 2020;12(4):4323‐4332. 31899611 10.1021/acsami.9b21296

[45] Hu Z , Wang G , Zhang R , et al. Sustained‐release behavior and the antitumor effect of charge‐convertible poly(amino acid)s drug‐loaded nanoparticles. Drug Deliv Transl Res. 2023;13(9):2394‐2406. 36913103 10.1007/s13346-023-01323-w

[46] Duro‐Castano A , Gallon E , Decker C , Vicent MJ . Modulating angiogenesis with integrin‐targeted nanomedicines. Adv Drug Deliv Rev. 2017;119:101‐119. 28502767 10.1016/j.addr.2017.05.008

[47] Uzel E , Durgun ME , Esentürk‐Güzel İ , Güngör S , Özsoy Y . Nanofibers in ocular drug targeting and tissue engineering: their importance, advantages, advances, and future perspectives. Pharmaceutics. 2023;15(4):1062. 37111550 10.3390/pharmaceutics15041062 PMC10145046

[48] Agrawal G , Aswath S , Laha A , Ramakrishna S . Electrospun nanofiber‐based drug carrier to manage inflammation. Adv Wound Care. 2023;12(9):529‐543. 10.1089/wound.2022.0043 36680757

[49] Priya S , Batra U , Samshritha RN , Sharma S , Chaurasiya A , Singhvi G . Polysaccharide‐based nanofibers for pharmaceutical and biomedical applications: a review. Int J Biol Macromol. 2022;218:209‐224. 35872310 10.1016/j.ijbiomac.2022.07.118

[50] Maccubbin DL , Cohen SA , Ehrke MJ . Indomethacin modulation of adriamycin‐induced effects on multiple cytolytic effector functions. Cancer Immunol Immunother. 1990;31(6):373‐380. 2386983 10.1007/BF01741409 PMC11038739

[51] Koch R , Aung T , Vogel D , et al. Nuclear trapping through inhibition of exosomal export by indomethacin increases cytostatic efficacy of doxorubicin and pixantrone. Clin Cancer Res. 2016;22(2):395‐404. 26369630 10.1158/1078-0432.CCR-15-0577

[52] Lérida‐Viso A , Estepa‐ Fernández A , Morellá‐Aucejo Á , et al. Pharmacological senolysis reduces doxorubicin‐induced cardiotoxicity and improves cardiac function in mice. Pharmacol Res. 2022;183:106356. 35843569 10.1016/j.phrs.2022.106356

[53] Yn Q , Gao R , Wei X , et al. Gasdermin D mediates endoplasmic reticulum stress via FAM134B to regulate cardiomyocyte autophagy and apoptosis in doxorubicin‐induced cardiotoxicity. Cell Death Dis. 2022;13(10):901. 36289195 10.1038/s41419-022-05333-3 PMC9606128

[54] Singal PK , Iliskovic N . Doxorubicin‐induced cardiomyopathy. N Engl J Med. 1998;339(13):900‐905. 9744975 10.1056/NEJM199809243391307

[55] Luo J‐Q , Liu R , Chen F‐M , et al. Nanoparticle‐mediated CD47‐SIRPα blockade and calreticulin exposure for improved cancer chemo‐immunotherapy. ACS Nano. 2023;17(10):8966‐8979. 37133900 10.1021/acsnano.2c08240

[56] You Q , Fang T , Yin X , et al. Serum CD4 is associated with the infiltration of CD4^+^T cells in the tumor microenvironment of gastric cancer. J Immunol Res. 2021;2021:1‐13. 10.1155/2021/6539702 PMC8246328 34258299

[57] Kravtsov DS , Erbe AK , Sondel PM , Rakhmilevich AL . Roles of CD4+ T cells as mediators of antitumor immunity. Front Immunol. 2022;13:972021. 36159781 10.3389/fimmu.2022.972021 PMC9500154

[58] Homet Moreno B , Zaretsky JM , Garcia‐Diaz A , et al. Response to programmed cell death‐1 blockade in a murine melanoma syngeneic model requires costimulation, CD4, and CD8 T cells. Cancer Immunol Res. 2016;4(10):845‐857. 27589875 10.1158/2326-6066.CIR-16-0060 PMC5050168

[59] Lee HH , Jung J , Moon A , Kang H , Cho H . Antitumor and anti‐invasive effect of apigenin on human breast carcinoma through suppression of IL‐6 expression. Int J Mol Sci. 2019;20(13):3143. 31252615 10.3390/ijms20133143 PMC6651620

[60] Kang S , Tanaka T , Narazaki M , Kishimoto T . Targeting interleukin‐6 signaling in clinic. Immunity. 2019;50(4):1007‐1023. 30995492 10.1016/j.immuni.2019.03.026

[61] Felcher CM , Bogni ES , Kordon EC . IL‐6 cytokine family: a putative target for breast cancer prevention and treatment. Int J Mol Sci. 2022;23(3):1809. 35163731 10.3390/ijms23031809 PMC8836921

[62] Chen H , Guo Q , Chu Y , et al. Smart hypoxia‐responsive transformable and charge‐reversible nanoparticles for the deep penetration and tumor microenvironment modulation of pancreatic cancer. Biomaterials. 2022;287:121599. 35777332 10.1016/j.biomaterials.2022.121599

[63] Liu Y , Huo Y , Yao L , et al. Transcytosis of nanomedicine for tumor penetration. Nano Lett. 2019;19(11):8010‐8020. 31639306 10.1021/acs.nanolett.9b03211

[64] Cabral H , Matsumoto Y , Mizuno K , et al. Accumulation of sub‐100 nm polymeric micelles in poorly permeable tumours depends on size. Nat Nanotechnol. 2011;6(12):815‐823. 22020122 10.1038/nnano.2011.166

[65] He S , Gou X , Zhang S , et al. Nanodelivery systems as a novel strategy to overcome treatment failure of cancer. Small Methods. 2024;8(1):e2301127. 37849248 10.1002/smtd.202301127

