# Supplementary material for: Matrix metalloproteinase 2‐responsive dual‐drug‐loaded self‐assembling peptides suppress tumor growth and enhance breast cancer therapy
Source: Bioeng Transl Med. 2024 Jul 17;9(6):e10702. doi: 10.1002/btm2.10702 (PMC11558207; doi:10.1002/btm2.10702)
Supplement: Supplementary file 1 — Data S1. Supporting information. [file BTM2-9-e10702-s001.docx]

Supporting Information for

**Matrix metalloproteinase 2-responsive dual-drug-loaded self-assembling peptides suppress tumor growth and enhance breast cancer therapy**

Jihong Ma ^a, †^, Haiyan Yang ^b, †^, Xue Tian ^c, †^, Fanhu Meng ^d^, Xiaoqing Zhai ^a^, Aimei Li ^d^, Chuntao Li ^d^, Min Wang ^d,^ *, Guohui Wang ^d,^ *, Chunbo Lu ^d,^ *, Jingkun Bai ^d,^ *

^a^ School of Clinical Medicine, Shandong Second Medical University, Weifang, 261053, China.

^b^ Yantaishan Hospital Affiliated to Binzhou Medical University, Yantai, 264003, China.

^c^ School of Basic Medical Sciences, Shandong Second Medical University, Weifang, 261053, China.

^d^ School of Bioscience and Technology, Shandong Second Medical University, Weifang, 261053, China.

† These authors contributed equally to this work.

* Corresponding authors.

E-mail addresses: jkbai@sdsmu.edu.cn (J. Bai); lcb@sdsmu.edu.cn (C. Lu); wangguohui1983@163.com (G. Wang); minwang@sdsmu.edu.cn (M. Wang)

**

**

**FIGURE S1** Structure of Pep1.





**FIGURE S2** Structure of Pep2.

**
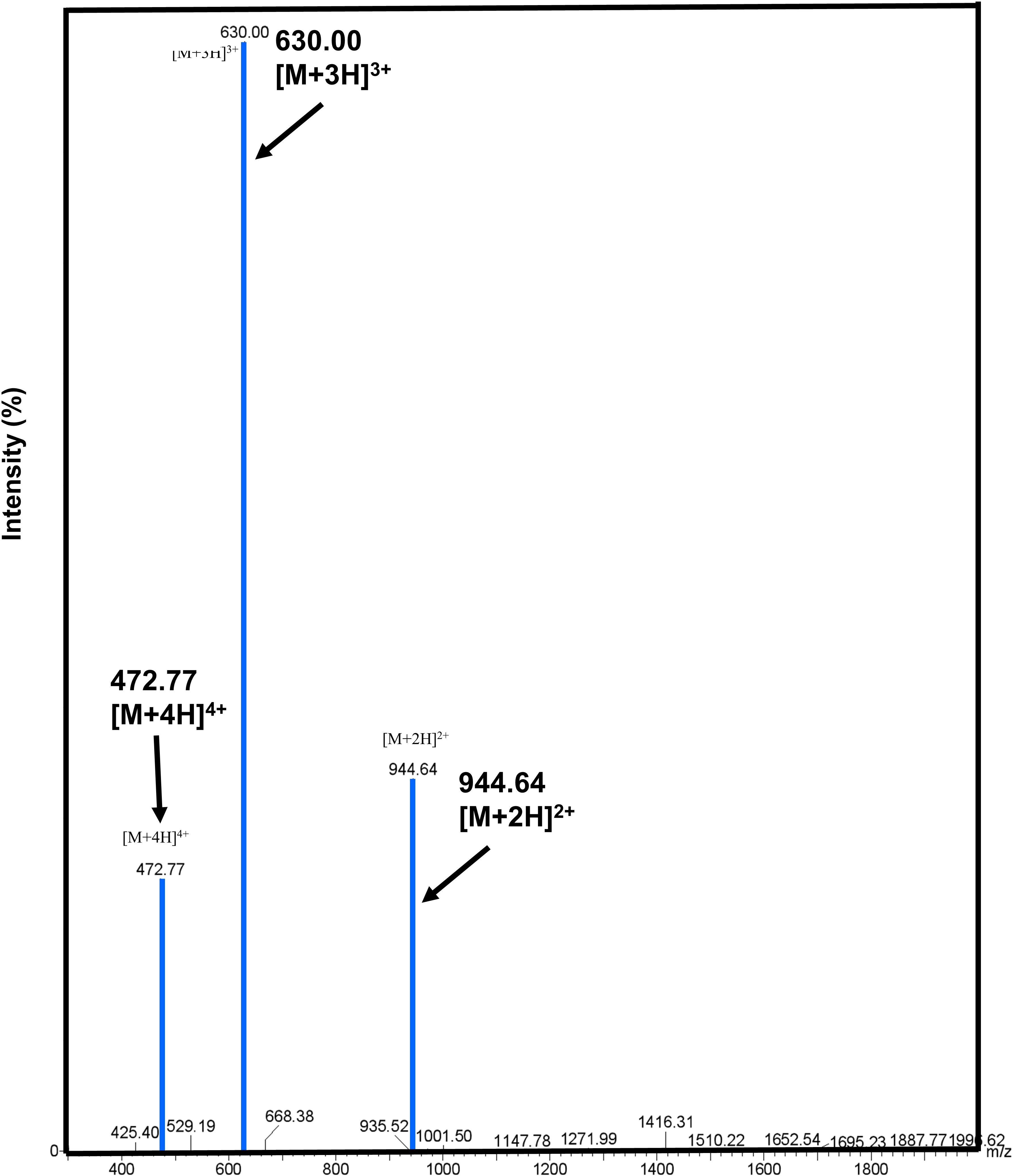
**

**FIGURE S3** MS of Pep1.

**
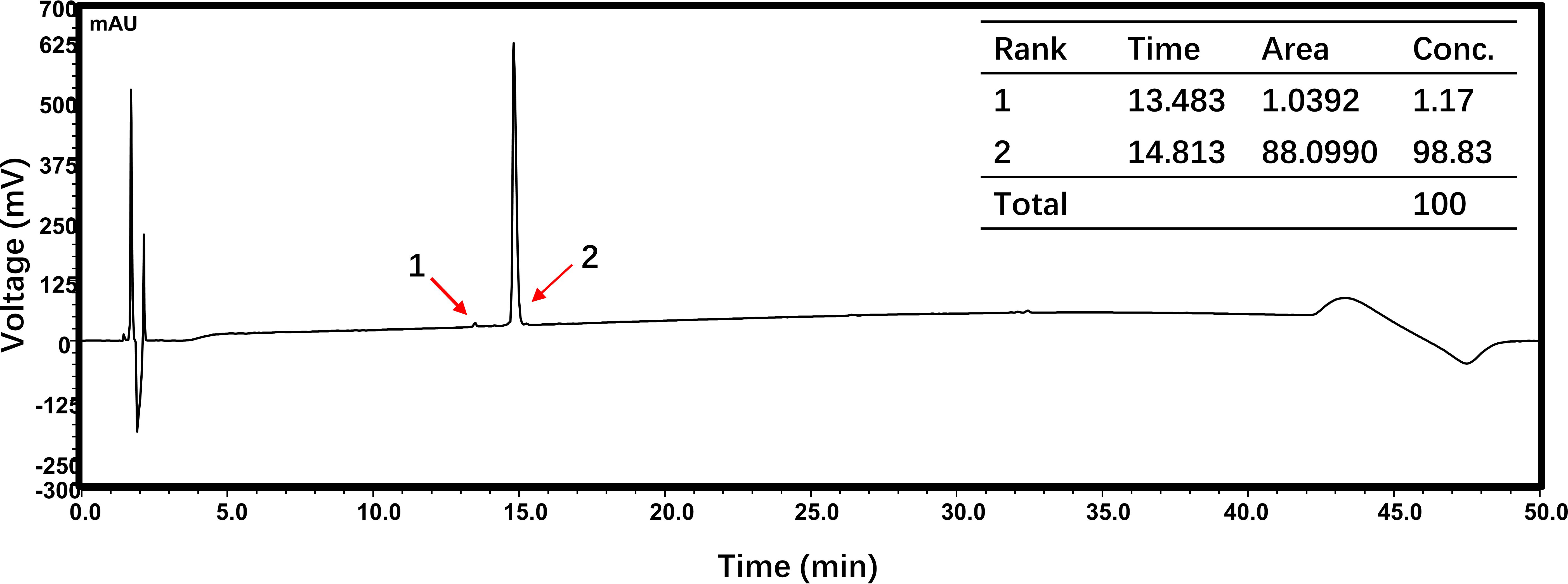
**

**FIGURE S4** HPLC chromatogram of Pep1.

**
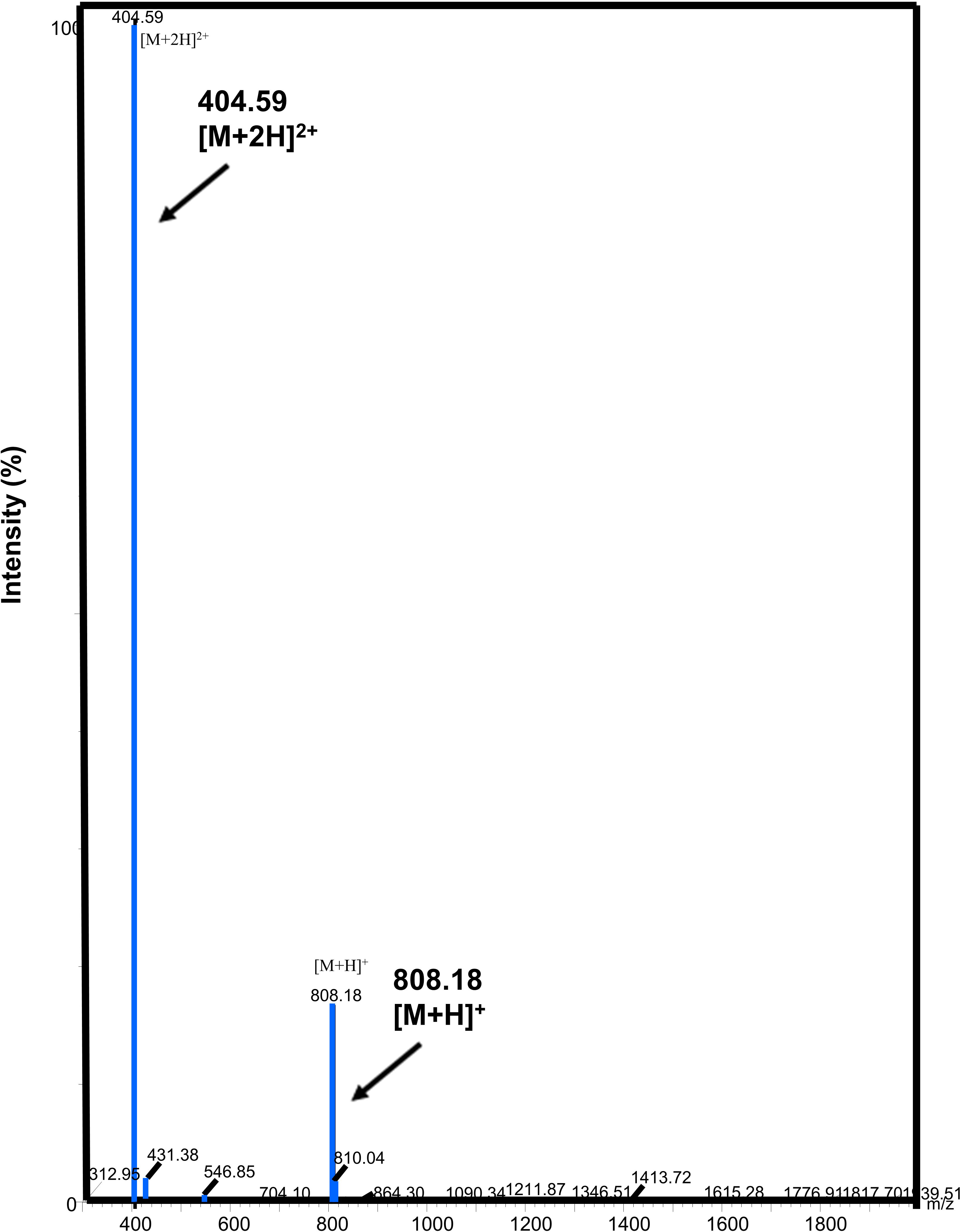
**

**FIGURE S5** MS of Pep2.


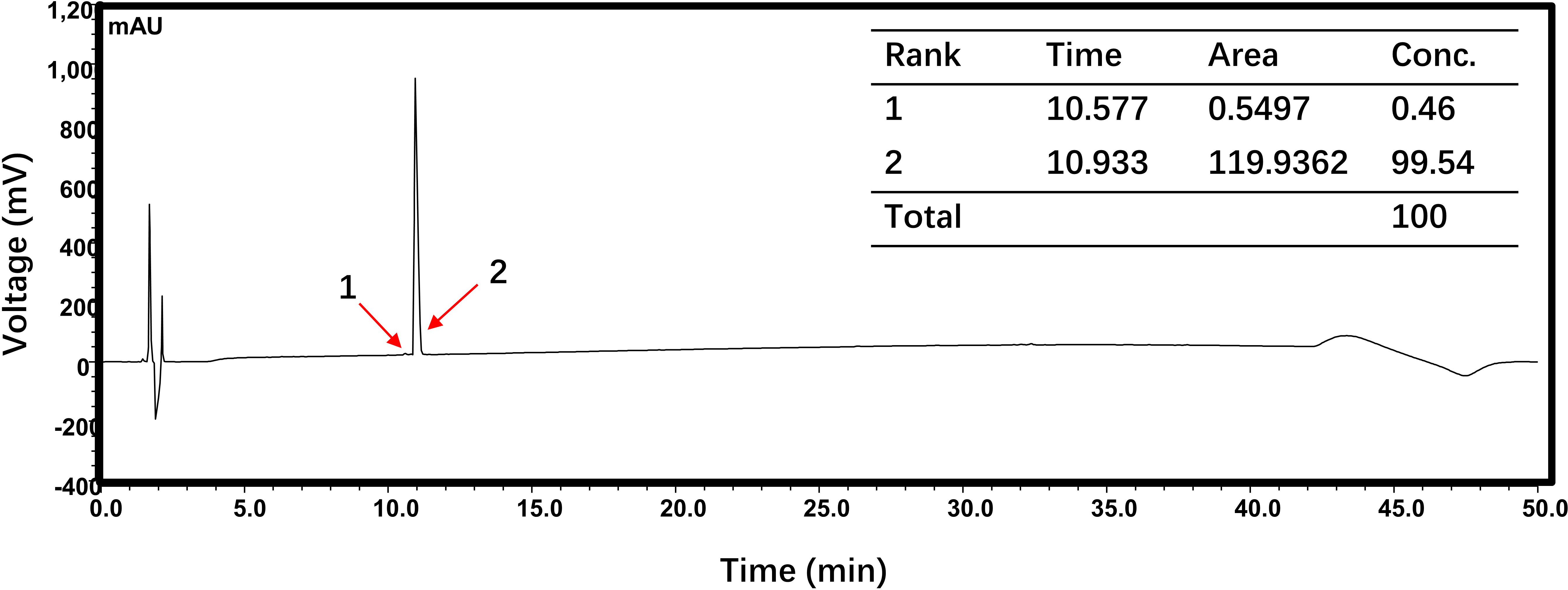


**FIGURE S6** HPLC chromatogram of Pep2.


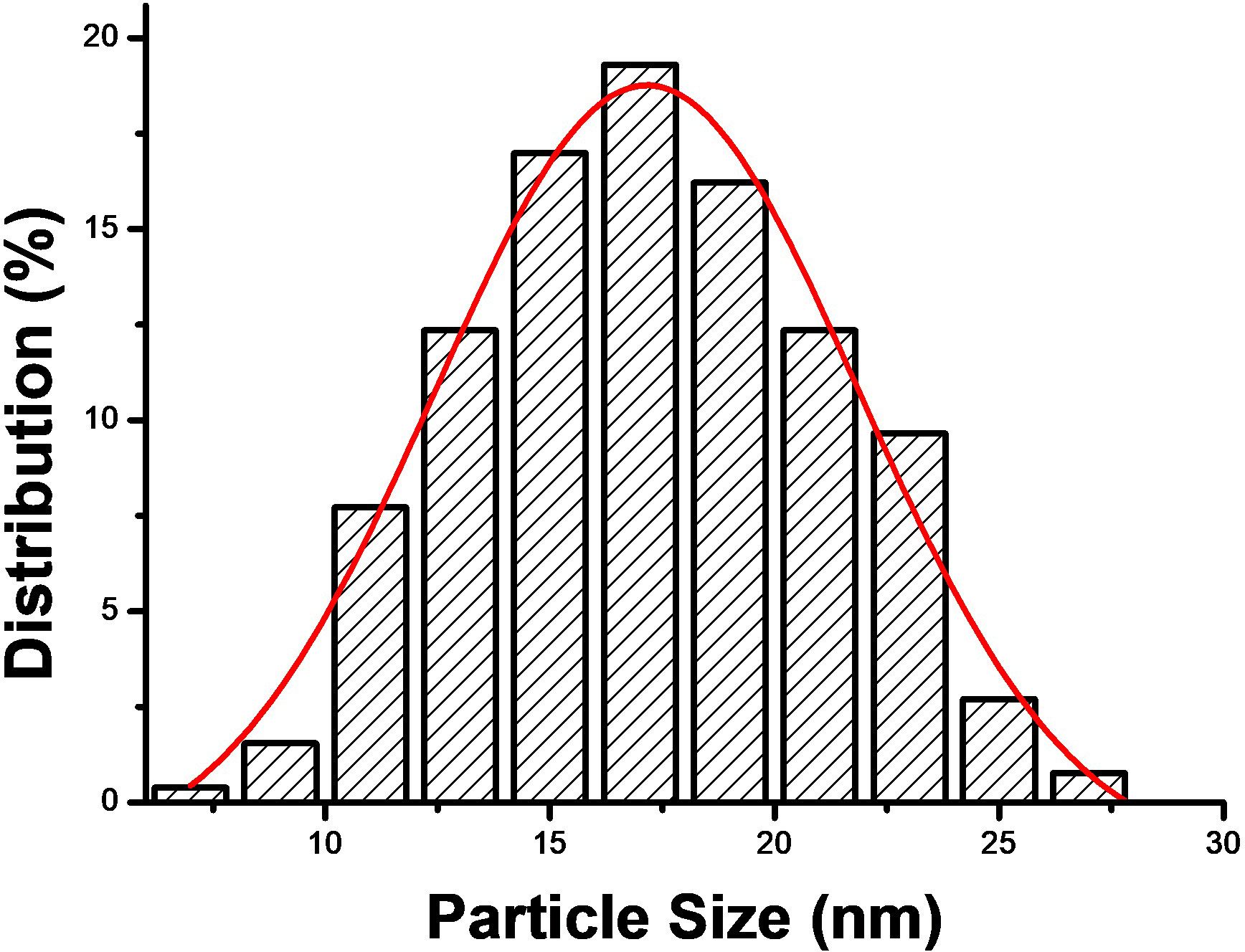


**FIGURE S7** Particle size distribution of Pep1.


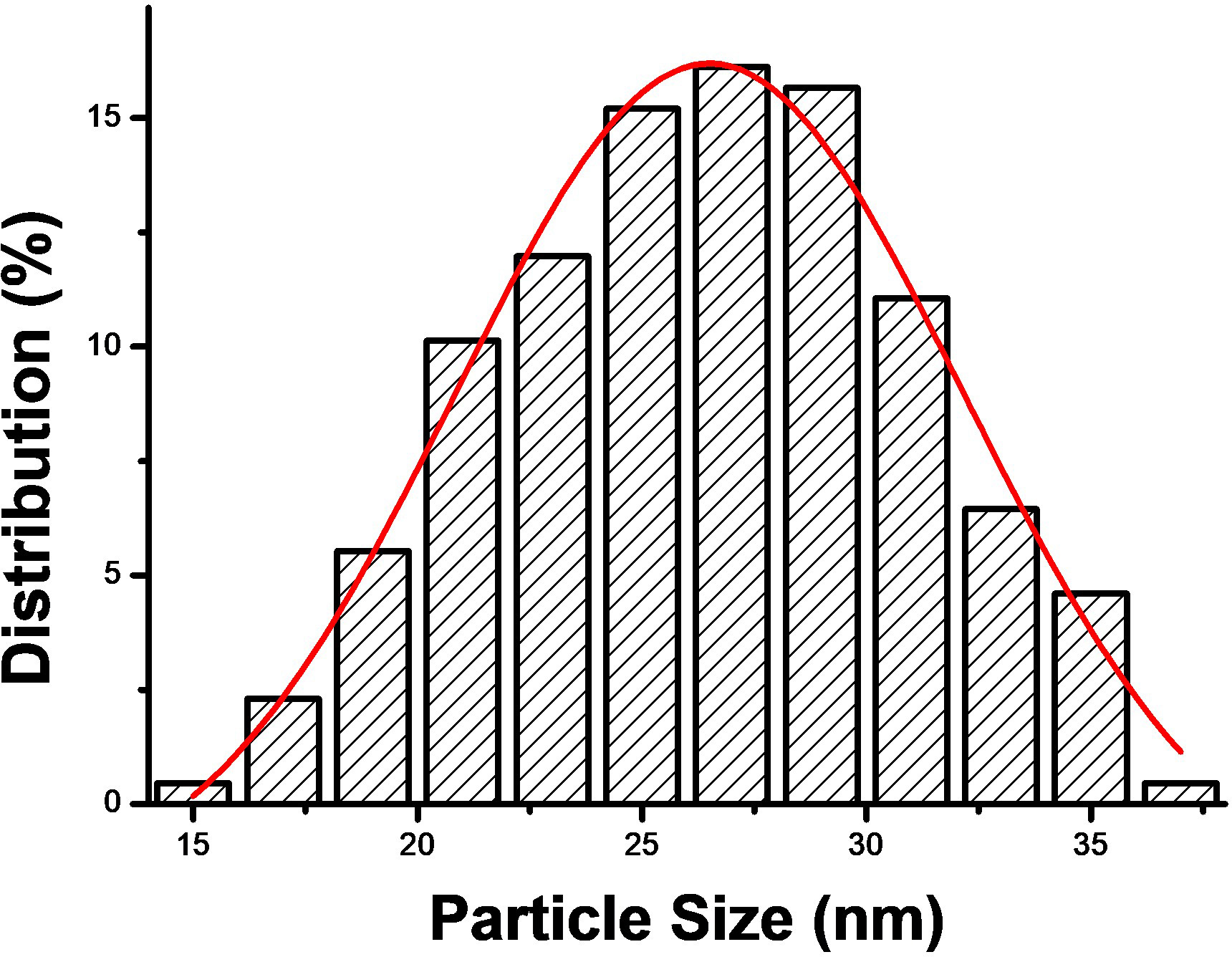


**FIGURE S8** Particle size distribution of DI/Pep1.


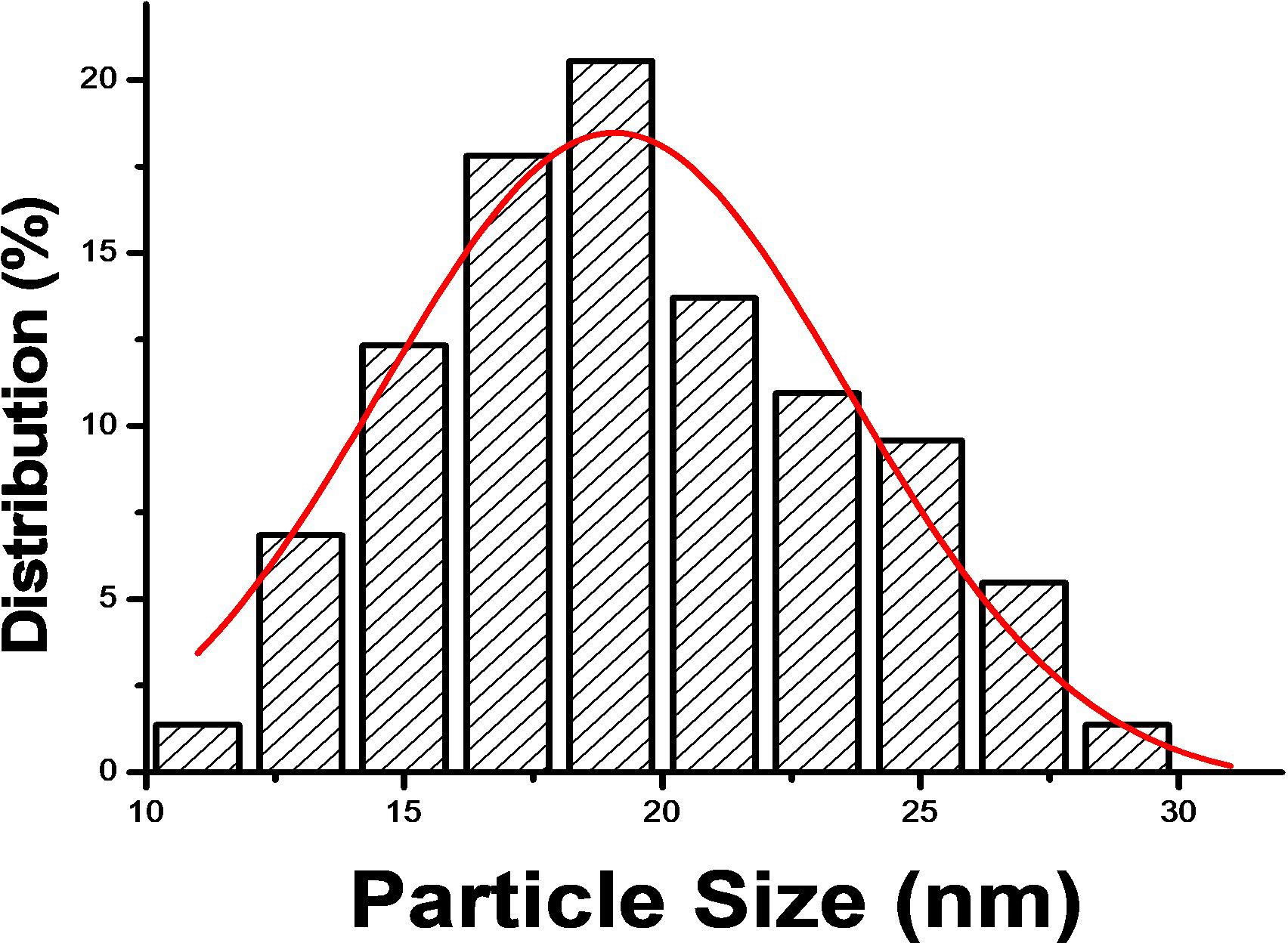


**FIGURE S9** Particle size distribution of Pep2.


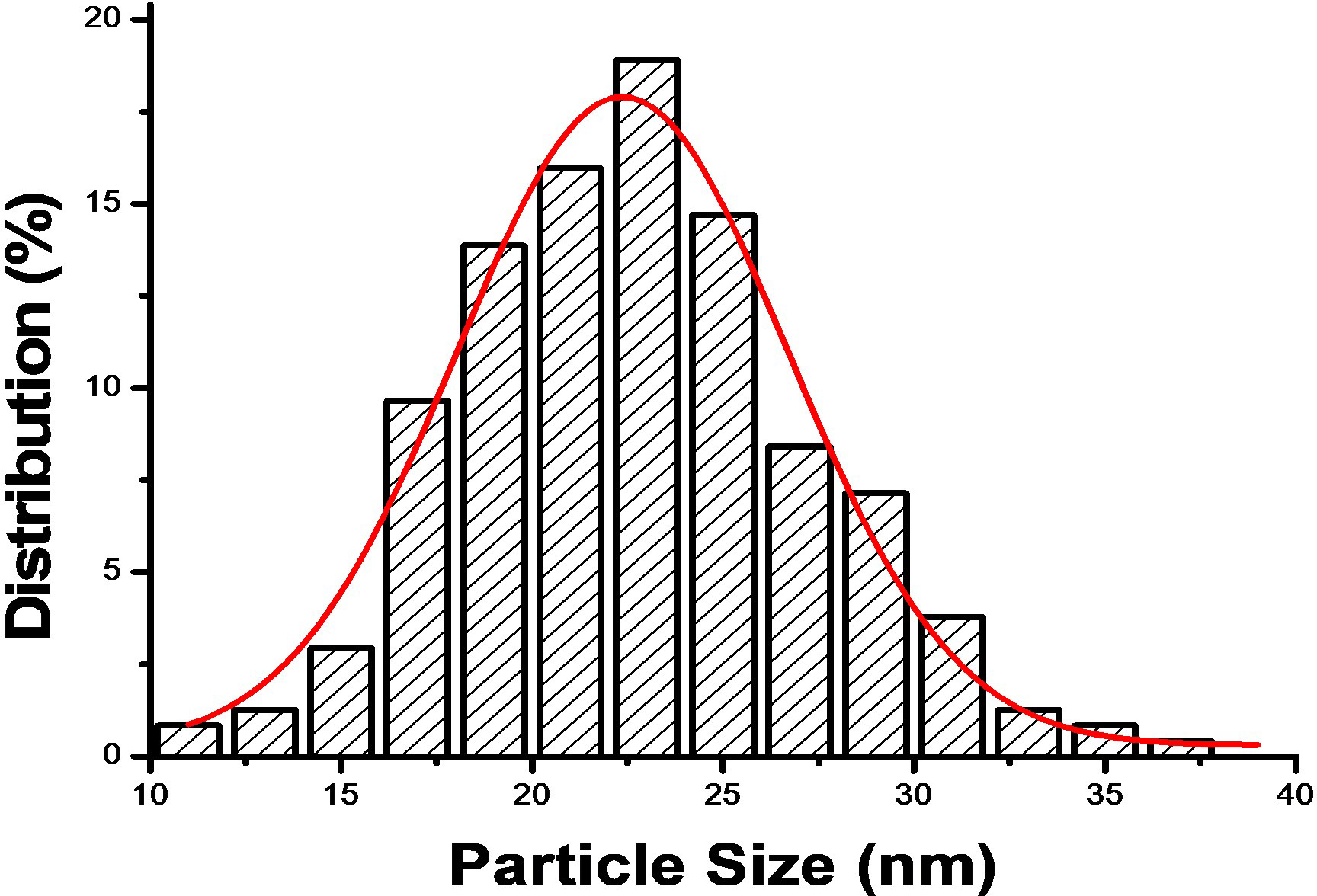


**FIGURE S10** Particle size distribution of DI/Pep2.


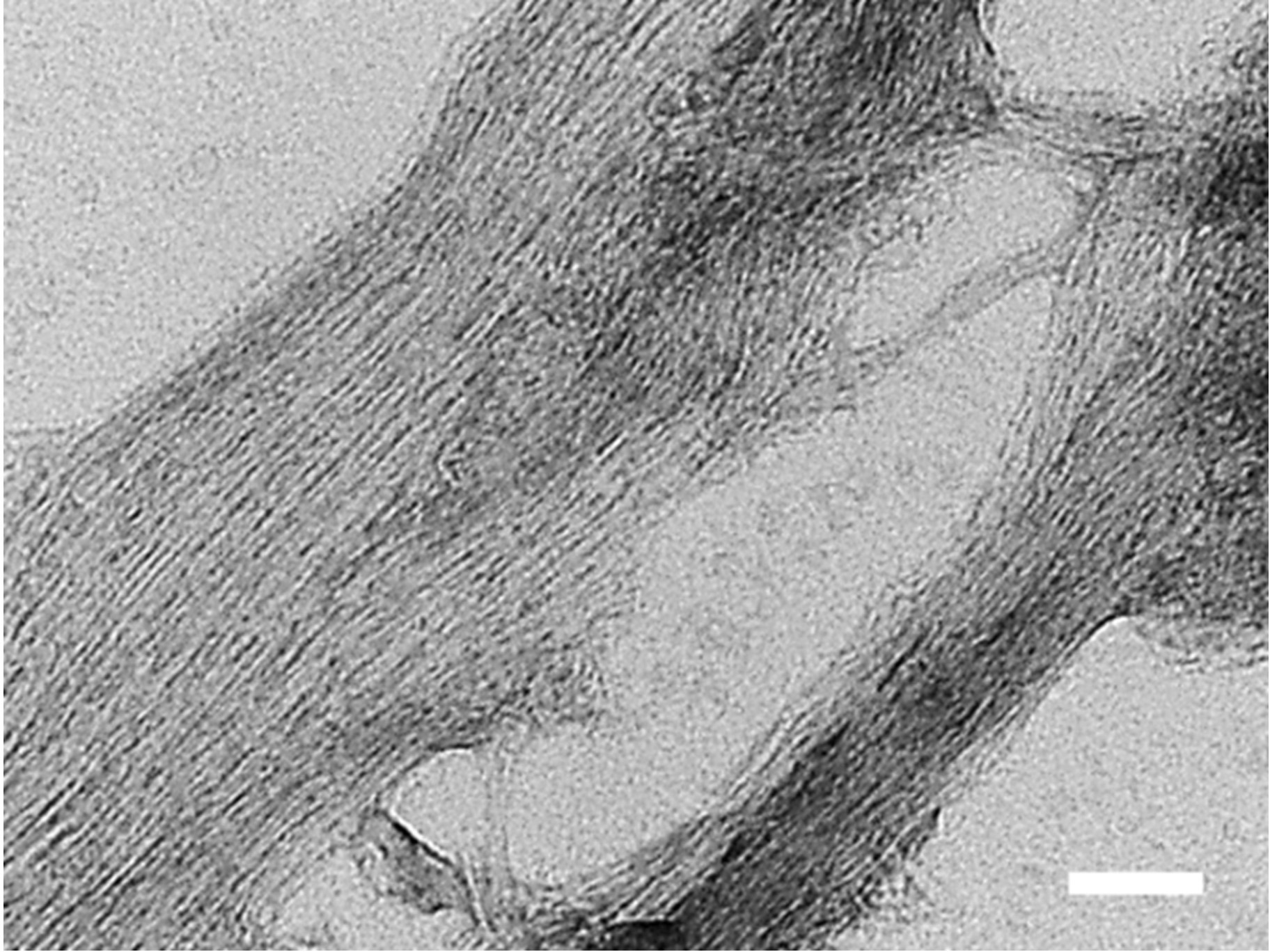


**FIGURE S11** DI/Pep1 TEM image after adding MMP-2. Scale bar: 100 nm.


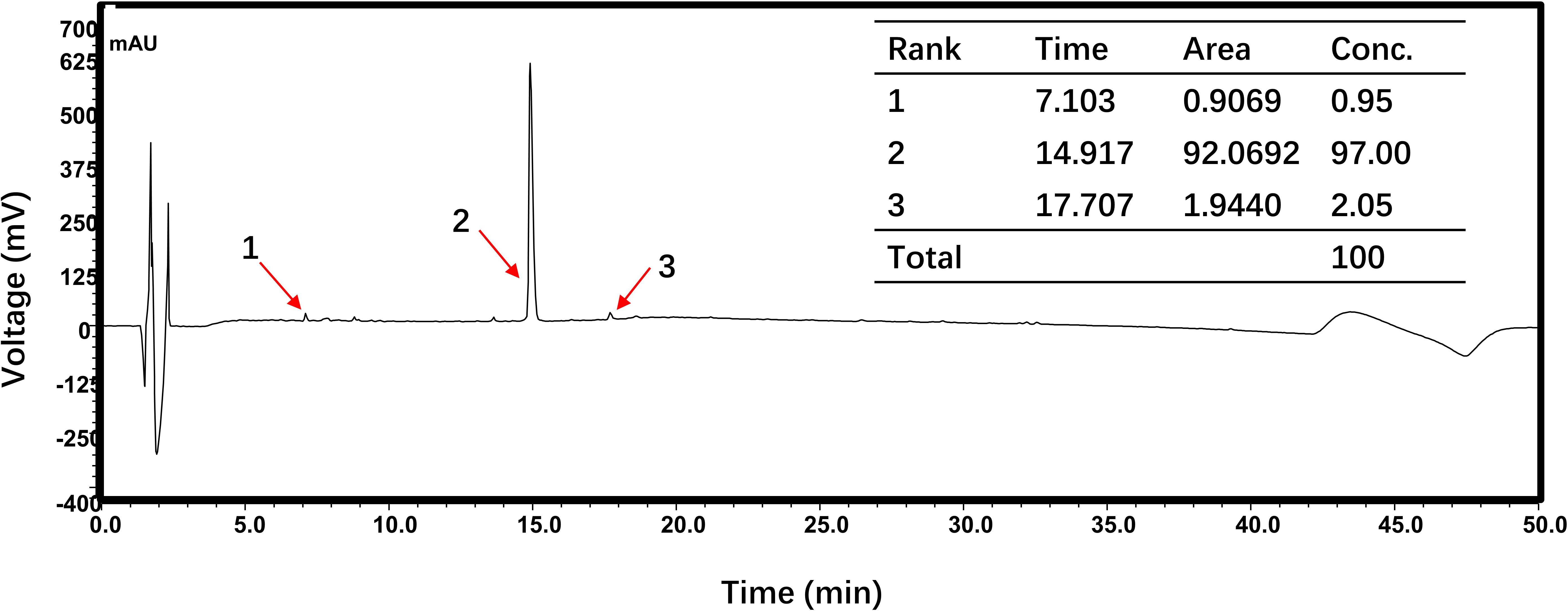


**FIGURE S12** HPLC chromatogram of Pep1 after the addition of MMP-2 for 24 h at 37 °C.
